# Nuclear and Chloroplast Markers Provide New Insights Into the Syngameon Dynamics of Genus *Micromeria* (Lamiaceae) in the Canary Islands

**DOI:** 10.1002/ece3.71843

**Published:** 2025-08-02

**Authors:** Manuel Curto, Pamela Puppo, Harald Meimberg

**Affiliations:** ^1^ CIBIO‐InBIO, Research Center in Biodiversity and Genetic Resources University of Porto Vairão Portugal; ^2^ BIOPOLIS Program in Genomics, Biodiversity and Land Planning CIBIO Vairão Portugal; ^3^ Institute of Integrative Nature Conservation Research, Department of Integrative Biology and Biodiversity Research BOKU University Vienna Austria; ^4^ Department of Biological Sciences Marshall University Huntington West Virginia USA

**Keywords:** genotyping by amplicon sequencing, hybridization, introgression, Macaronesia, migration

## Abstract

Species syngameons are groups of more than two hybridizing species that form complex hybrid networks. Syngameons facilitate sharing the gene pool among species while maintaining morphological differentiation. In oceanic islands, hybridization is common, and syngameons are expected to be common and play an important role in increasing standing variation in the face of the founder effect associated with the colonization process. The mechanisms of how these syngameons are formed and maintained, the impact islands' geological history has on syngameons, and their evolutionary consequences remain unknown. Using the genus *Micromeria* (Lamiaceae) in the Canary Islands as an example, we aim to describe the structure of the syngameons and evaluate if it varies across island age, taxa, and genomic region. For this, we used 14 Exon primed intron spanning (EPIC) nuclear markers and 12 chloroplast (cpDNA) markers to conduct phylogenetic and genetic diversity analyses. The results show that species in younger islands have higher genetic diversity and share haplotypes with more taxa than species in older islands. Moreover, widespread taxa have higher intraspecific connectivity than taxa with narrower distributions. These findings suggest that species syngameons are larger and more complex in younger islands and that widespread taxa are key players in maintaining them. This pattern and phylogenetic signal were not consistent across loci and genomic compartments, indicating that different genomic regions may show different perspectives on syngameons dynamics. This study provides evidence that island ontogeny, degree of evolutionary divergence, and species distribution range shape the formation, expansion, and maintenance of syngameons.

## Graphical abstract

This study explores species syngameons, hybridizing networks that facilitate gene pool sharing while maintaining species differentiation, using *Micromeria* in the Canary Islands as a model. The findings reveal that syngameons are larger and more complex on younger islands, with widespread taxa playing a critical role in maintaining these networks. The results also highlight variability in syngameon patterns across genomic regions, suggesting that island age, evolutionary divergence, and species distribution influence syngameon formation and dynamics.

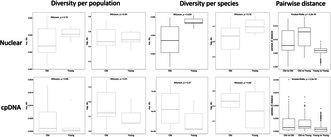

## 1 Introduction

Traditionally, hybridization was thought to be restricted to a few taxa, mostly plants, with little to no impact in generating new diversity (Barton 2013; Taylor and Larson 2019). This view changed with the availability of molecular markers. In particular, with the appearance of high‐throughput sequencing techniques, researchers gathered evidence of both recent and ancient hybridization to be widespread across the tree of life (Abbott et al. 2016; Moran et al. 2021). In fact, hybrids are expected to be found in 25% of plants and 10% of animals (Mallet 2005), and the role of hybridization in generating new diversity is becoming obvious (Irisarri et al. 2018; Osborne et al. 2016). This encompasses processes like homoploid and allopolyploid hybrid speciation, where a hybrid population becomes reproductively isolated from the parental species (Abbott et al. 2013), but also introgression resulting from hybridization and backcrossing to one of the parental species (Leroy et al. 2020). This can lead to the gain of new traits permitting species to explore new ecological niches (Leroy et al. 2020). These processes played a crucial role in some of the most impressive known radiation events (Meier et al. 2017) and should be thoroughly explored.

When more than two species are interlinked by gene flow and introgression, they form a syngameon, a network of multiple species that can hybridize with each other (Boecklen 2017; Buck and Flores‐Rentería 2022; Lotsy 1925). Several examples of syngameons have been described, including, the American white oaks (Hardin 1975), the European *Populus* L. species (Chhatre et al. 2018), and the genus *Potamogeton* L. (Clapham et al. 1990). However, many other cases might have been overlooked, especially in groups with a complex evolutionary history, as these are generally understudied. Likewise, the mechanism of how these syngameons are formed and maintained, and their evolutionary consequences, remains unknown although some hypotheses are outlined (Buck and Flores‐Rentería 2022). For example, Liu et al. (2017) and Seehausen (2004) postulate that syngameons are more likely to be formed among taxa resulting from rapid radiations since barriers to gene flow were not given time to fully establish. Cronk and Suarez‐Gonzalez (2018) propose that range shifts can promote the formation of species syngameons through the establishment of secondary contact of previously isolated species. By analyzing the structure of several syngameons, Boecklen (2017) concluded that geographically widespread taxa tend to be the main contributors to the syngameon because they are more likely to form contact zones with more species where gene flow can occur. Despite the complex network of interspecific gene flow that is established in these systems, taxa can still maintain morphological identity through a combination of purifying selection and recombination that purges foreign variation and keeps neutral or advantageous variants (Cannon and Lerdau 2015). Consequently, there is a proportion of the species gene pool that can be shared across the syngameon, which has been hypothesized to benefit rare species since it reduces the chances of inbreeding depression (Cannon and Lerdau 2015).

Plant taxa on oceanic islands are frequently isolated through ecological barriers rather than through intrinsic barriers to gene flow, making hybridization common (Crawford and Archibald 2017). Hybridization is further promoted by the highly dynamic nature of these systems where both natural and anthropogenic disruption is common, and many times brings previously isolated species into contact (Kerbs et al. 2017). In this context, islands can be expected to also have a relatively high prevalence of synagemons, and their formation and shape should be influenced by the oceanic islands' dynamic nature (Caujapé‐Castells et al. 2017). The latter results from two general forces: volcanic activity that increases the island's area and height profile, and erosion that creates heterogeneous topography at first, but then makes the island sink back below the ocean's surface (Jackson 2013). The general dynamic model of island biogeography takes these processes into consideration when explaining fluctuations of species richness through time (Whittaker et al. 2008, 2010). This model states that colonization and speciation rates increase with the island's gain in area and topographic complexity, while the extinction rate is favored by the loss of altitudinal range.

The significant role that species syngameon formation has on shaping the island's biodiversity was first proposed by Caujapé‐Castells et al. (2011) by outlining the surfing syngameon hypothesis (SSH) which links the concept of syngameon to the dynamic nature of oceanic islands and their geological evolution. This hypothesis was further developed in Caujapé‐Castells et al. (2017) by incorporating island geomorphological dynamics. The SSH not only predicts volcanic activity and erosion as processes causing and shaping syngameons but also the impact they have on genetic diversity. The SSH is based on two observations on plants in the Canary Islands: (1) the high incidence of hybridization and (2) the fact that, when compared with their mainland counterparts, island taxa showed higher genetic diversity than expected considering the founder effect (García‐Verdugo et al. 2015). The SSH postulates that the emergence of new islands opens the opportunity for previously isolated lineages to meet and form gene flow connections and consequently new syngameons. This process can happen multiple times and is promoted by the increase of island area and height through volcanic activity. This increase in island topographic complexity promotes speciation and the establishment of barriers to gene flow, breaking the syngameon. The establishment and expansion of the syngameon through multiple colonization events of an island counteracts founder effects since it increases the chance of bringing genetic diversity from the source. Moreover, this diversity can be recombined through hybridization, forming new variation. The differentiation process compartmentalizes genetic diversity and decreases taxon effective population size, making them more vulnerable to drift. Consequently, genetic diversity is expected to be higher in younger islands, while genetic differentiation is higher in older islands. Curto et al. (2017) confirmed this expectation on the genus *Micromeria* in the Canary Islands. Moreover, similar patterns were observed during the expansion/colonization of *Micromeria* and within the same island as a response to volcanic activity (Puppo et al. 2016). Furthermore, Curto et al. (2018) found evidence of ancestral introgression that correlates with ecological conditions, supporting the adaptive value of gene flow within a syngameon. These findings support not only the evolutionary impact of the syngameon during island colonization but also its dynamic nature, providing different evolutionary opportunities in the course of its development. To investigate how the syngameon structure unfolds over time, the context of islands geodynamics is particularly suitable.

The SSH has been outlined having the geological dynamics of the Canary Islands in mind. This archipelago was formed by a volcanic hotspot moving from east to west, leaving older islands in the east and younger islands in the west (Fernández‐Palacios et al. 2011). The islands' age ranges between 20.6 million years ago (Mya) in Fuerteventura and 1.1 Mya in El Hierro. Islands are in different stages of their ontogeny, and therefore different forces shape their biodiversity (Fernández‐Palacios et al. 2011). In the east, erosion is the main process shaping the islands' geomorphology, with speciation and extinction being the main hypothesized evolutionary forces. Conversely, in the west, volcanic activity is frequent, and islands are still gaining height and topographic complexity, meaning colonization is expected to be more prevalent. In the context of the SSH, inter‐species connections in the syngameons are expected to be more interrupted in the east than in the west, where new hybrid connections should foster the syngameon dynamics. Consequently, genetic diversity and shared genetic variation are expected to be higher in the west than in the east.

The Canary Islands are one of the centers of diversity of the genus *Micromeria* with 22 endemic species (Puppo et al. 2015, 2023). The diversification of this genus in the archipelago is estimated to have started 8.4 Mya with the formation of two lineages, one found mostly in the eastern islands and the other in the western islands (Puppo et al. 2014, 2015). Gran Canaria and Tenerife are the centers of diversity of the eastern and western lineages, with seven and nine species, respectively. These species form paraphyletic groups with the remaining species of the archipelago, indicating that they are likely the source of colonization of the remaining islands (Puppo et al. 2015). The eastern lineage, besides the taxa found in Gran Canaria, includes *M. mahanensis* Puppo from Lanzarote and Fuerteventura, and *M. lepida* Webb & Berthel. and *M. gomerensis* (P.Pérez) Puppo from La Gomera. The western lineage includes taxa found in the remaining islands and Madeira, as well as *M. pedro‐luisii* Puppo from La Gomera. In this scope, La Gomera is quite unique since it was colonized by both lineages (Curto et al. 2017; Puppo et al. 2015). Considering that Fuerteventura and Lanzarote constitute the same volcanic edifice, all species are single‐island endemics. *Micromeria* species have different distribution ranges. Some taxa grow on most of the island where they occur (like *M. ericifolia* (Roth) Bornm. in Tenerife or *M*. *herpyllomorpha* Webb & Berthel. in La Palma), while other taxa are restricted to small stretches of land (like the narrow endemics *M. rivas‐martinezii* Wildpret and *M. glomerata* P.Pérez in Tenerife). The radiation of the species of *Micromeria* is thought to be fueled by both ecological and geological factors (Meimberg et al. 2006; Puppo et al. 2014, 2015). *Micromeria* species are considered entomophilous, with several species of Diptera and Hymenoptera having been observed visiting the flowers during fieldwork in Gran Canaria and La Gomera (Puppo, personal observation), though at this point, the lack of studies focusing on pollinators makes the role of pollinators unclear in the speciation process of the group.

Both Curto et al. (2017) and Puppo et al. (2016) found high levels of admixture and significant interspecific gene flow among species of *Micromeria* in the Canary Islands, which, together with field observations of a wide range of morphological hybrid forms, strongly indicates the existence of a syngameon dynamics. However, so far, this dynamics has only been explored using genotype‐based approaches like microsatellites (e.g., Curto et al. 2017; Puppo et al. 2016). Although these markers provide information about the distribution patterns of genetic diversity and differentiation, they are less suitable for retrieving phylogenetic relationships between the involved species, which may be a key factor in understanding syngameons' dynamics. The phylogenetic studies of *Micromeria* have included multiple individuals per species but were not designed to investigate different populations and individuals within the same population that may contribute differently to the syngameon structure. Recently, amplicon sequencing started to be used for genotyping purposes, producing simultaneously sequence data that can be used in a phylogenetic context and genotypic data for frequency‐based analyses (Curto et al. 2019; Tibihika et al. 2020). This method is known as genotyping by amplicon sequencing (GBAS) and it can produce data for high sample sizes, making it ideal for population‐based studies (Tibihika et al. 2020). GBAS can be combined with established chloroplast and nuclear exon‐primed intron‐spanning (EPIC) markers that can uncover phylogenetic relationships at multiple levels (Li et al. 2010; Thomson et al. 2010). Such dataset allows combining frequency‐based and phylogenetic analysis to show patterns of haplotype distribution across species and their relationship to each other. Furthermore, diversity measures combining sequence identity and frequency information can be calculated, so an increase in diversity resulting from syngameons combining highly divergent haplotypes can be detected (Sweigart and Willis 2003).

In the current study, we use amplicon sequencing to genotype nuclear (EPIC) and chloroplast markers to investigate the syngameon dynamics of *Micromeria* in the Canary Islands. We utilize the duality of this approach by using both sequence identity and allele frequency information to describe genetic diversity, differentiation, haplotype sharing, and phylogenetic patterns across islands, and the main lineages and species of this genus. Specifically, our study aims to: (1) describe the structure of the *Micromeria* syngameons in the Canary Islands; (2) evaluate how they relate to island age; and (3) test the role that each taxon has in maintaining the syngameons. This work should bring new insights on the role of hybridization during the diversification of plants in oceanic islands.

## 2 Material and Methods

### 2.1 Nuclear Markers Genotyping

The EPIC nuclear markers developed by Curto et al. (2012) were redesigned to fit the amplicon sequencing framework, making them specific to the *Micromeria* species present in the Canary Islands and decreasing the maximum amplicon size to 580 bp. To this end, existing Lamiaceae sequences from 24 EPIC markers were downloaded from GenBank and aligned with the online version of the program mafft (Katoh et al. 2019). For some markers, there were no sequences of *Micromeria* available. During this study, we sequenced and assembled the genome of four *Micromeria* individuals (Table S1). These were based on shotgun sequencing libraries of an average insert size of 500 bp that were paired‐end sequenced on an Illumina HiSeq 2000 (Illumina, San Diego, California, USA) for 100 cycles each end. Library preparation and sequencing were performed by the University of Chicago Genomics Core facility. The resulting reads were quality controlled with Trimmomatic (Bolger et al. 2014) and used to assemble the genome using Abyss v.1.2.10 (Simpson et al. 2009) with a K‐mer size of 61 bp. Only contigs larger than 200 bp were kept. Genome completeness was assessed with BUSCO v5.7.1 (Simão et al. 2015) for the presence of core Eukaryote, Viridiplantae, and Eudicots single copy genes (Table S1). We used these to fill missing information by identifying homologous contigs based on megablast searches (Morgulis et al. 2008). The best match was retrieved and aligned together with the remaining sequences to define conserved motifs between 18 and 22 bp for primer design.

Primers were designed with a target melting temperature of 55°C, and the probability of formation of secondary structures was evaluated with primer3 (Untergasser et al. 2007). Primer sequences were extended with part of the Illumina adapters at the 5′ end to allow for amplicon sequencing library preparation. Each of the 24 designed primer pairs was then tested in vitro using three *Micromeria* samples (Table S2), one from the Canary Islands (*M. hierrensis* (P.Pérez) Puppo) and two from the mainland ( *M. inodora* (Desf.) Benth and *M. hochreutineri* Maire). Amplification was done in 10 μL reactions containing 5 μL of QIAGEN Multiplex PCR Master Mix (Qiagen, CA, USA), 4 μL of each primer (1 μM), and 1 μL of template/genomic DNA. PCR was conducted using the following temperature profile: 95°C for 15 min; 30 cycles of 95°C for 30 s, 55°C for 1 min, and 72°C for 1 min; and a final extension at 72°C for 10 min.

A total of 283 samples were included in this study, encompassing all 23 described species of *Micromeria* for the Canary Islands and Madeira, and three additional ones from the mainland to serve as outgroups (Table S2). All samples, and consequently DNA extracts, were taken from previous studies (Curto et al. 2017, 2018; Puppo et al. 2014, 2015, 2016).

Nuclear markers were genotyped in two multiplexes of 16 and 8 primer pairs (Table S3). Library preparation and genotyping were performed using the same protocol described in Curto et al. (2019). In short, the multiplexed primers were used to amplify the target regions following the same protocol and the initial test. The resulting PCR products were then purified using magnetic beads to remove dimers and unused primers. Finally, the PCR products were pooled per sample and used as a template for a second PCR reaction, in which index motifs were added to the amplicons. The resulting libraries were sequenced at the Genomics Service Unit of the Ludwig Maximilian University (Munich, Germany) as part of an Illumina MiSeq run (Illumina Inc., San Diego, California) producing 300 bp paired‐end reads.

Resulting sequences were analyzed with the SSR‐GBS‐pipeline (https://github.com/mcurto/SSR‐GBS‐pipeline) as described in Curto et al. (2019). This method determines genotypes in two steps. First, it identifies the most likely amplicon lengths under both diploid and haploid scenarios. Second, it assesses variation within each amplicon length. One of the outputs of this analysis consists of haplotype sequences per locus per sample that were used in downstream analyses organized in two ways. First, the phased information was kept for single locus‐based analyses. Second, both haplotypes were aligned and merged in a consensus sequence using IUPAC ambiguity codes if necessary. The latter was used for concatenated super matrix analyses. Markers and samples missing more than 50% of information were excluded.

### 2.2 Phylogenetic Analysis

Phylogenetic relationships were evaluated through both maximum likelihood and Bayesian inferences as implemented by the programs IQ‐TREE v3.0.0 (Nguyen et al. 2015) and MrBayes v3.2.7 (Ronquist et al. 2012). The alignments from the 14 nuclear markers were concatenated, resulting in a matrix of 6061 bp. Because phased information cannot be concatenated, a consensus sequence from both haplotypes was calculated. Substitution model selection was conducted using the program Partition Finder (Lanfear et al. 2017). For each marker, four types of partitions were defined: introns, and 1st, 2nd, and 3rd codon positions. All available models were tested, branch lengths were assumed to be linked, and model selection was based on the Akaike information criterion (Bozdogan 1987). Maximum likelihood analysis with IQ‐TREE ran using the available online tool W‐IQ‐TREE (Trifinopoulos et al. 2016). Branch support was obtained through 1000 ultrafast bootstrap replicates. To make time calibration possible (see description below), the maximum likelihood tree included two additional outgroups of the genera *Origanum* and *Mentha*. These were obtained through the *Mentha × piperita* (GCA_041146485.1) and *Origanum vulgare* (GCA_047713405.1) genomes available in GenBank. For MrBayes, the optimal model setting was then saved in a nexus file together with the input matrix. Tree search was done using two independent Markov Chain Monte Carlo runs, four chains each, that ran for 50 M generations, sampling every 1000th. Parameter convergence and run burnin values were evaluated using Tracer v1.7.1 (Rambaut et al. 2018). In the end, the first 10 M generations were excluded as burnin. The resulting tree was visualized and edited using Figtree (http://tree.bio.ed.ac.uk/software/figtree/).

Additionally, divergence times among the main lineages were estimated using the function chronos from the R package ape (Paradis et al. 2004). This was done with three calibration points spread across the topology to account for molecular clock saturation and heterogeneity throughout lineage divergence. The first point corresponded to the time of the most recent common ancestor (TMRCA) of the Menthinae subtribe (23 Mya) estimated by Drew and Sytsma (2012). The second and third calibration points corresponded to the TMRCA of all *Micromeria* of the Canary Islands (8.4 Mya) and all species growing on the island of Tenerife (6.7 Mya), which have been previously calculated by Puppo et al. (2014). Ranges of calibration points were calculated considering a normal distribution with a standard deviation of 2 Mya, and the chronos function was run using a relaxed model.

### 2.3 Genetic Diversity and Divergence Across Islands' Age

Genetic diversity was estimated both per population and per species. Only populations and species represented by at least five individuals were included. The average nucleotide diversity (*pi*) and haplotype diversity (*h*) across all nuclear markers were calculated using the R package *pegas* (Paradis 2010). Genetic diversity was compared between two island age categories (*Old* and *Young*) similar to Curto et al. (2017) using the criteria described there. The *Old* category included all species growing mostly in older parts of the archipelago that emerged more than 10 Mya and includes the islands of Lanzarote, Fuerteventura, Gran Canaria, and La Gomera. In Tenerife, there is a group of species (*M. gomerensis*, *M. rivas‐martinezii*, *M. densiflora* Benth., *M. teneriffae* (Poir.) Benth., and *M. tragothymus* Webb & Berthel.) that stem from an early divergent lineage (Puppo et al. 2014, 2015) and grow mostly in older parts of the island. These were also included in the *Old* category. The remaining species from Tenerife and the taxa from La Palma and El Hierro were included in the *Young* category. Since none of the genetic diversity statistics followed a normal distribution, significant differences between age categories were tested using the non‐parametric Wilcoxon signed‐rank test and visualized through boxplots in R (R Core Team 2023).

Patterns of genetic divergence between groups were tested using pairwise genetic distance across all individuals, resulting in three categories of comparison: *Young, Old*, and *Old* vs. *Young*. Divergence was calculated at the individual level, allowing us to use the full dataset including species and populations with few individuals. Pairwise distances were calculated under the Jukes‐Cantor model with the R package *ape* (Paradis et al. 2004). Differences between categories were tested using the nonparametric Kruskal‐Wallis test, the Wilcoxon signed‐rank test equivalent for more than two groups, and visualized through boxplots in R.

### 2.4 Admixture

As a proxy of admixture, patterns of shared haplotypes between species were evaluated by calculating the number of species that each taxon shares haplotypes with. We assumed that species sharing haplotypes with a higher number of species are more admixed than species sharing haplotypes with a lower number of species. Differences between *Old* and *Young* islands were tested and described for genetic diversity measures. We further explored patterns of haplotype sharing by conducting a minimum spanning haplotype networks approach as implemented in PopArt (http://popart.otago.ac.nz). Given that each marker individually is unlikely to contain enough resolution to differentiate among species of the same island or lineage, we evaluated if there were haplotypes shared between the eastern and western lineages.

Gene flow between species was assessed through the calculation of recent migration rates between species using BayesAss edition 3 (Mussmann et al. 2019). These calculations are based on allele frequency; thus, a codominant matrix based on whole sequence information, as described in Curto et al. (2019) was used. After inspecting convergence parameter values in Tracer v1.7.1 (Rambaut et al. 2018), the program ran for 25 M generations, saving results every 1000th, and the burnin value was set to 15 M. Mixing parameters for migration rates, allele frequencies, and inbreeding coefficient were set to 0.3, 0.7, and 0.7, respectively. Only migration rates with a minimum value of confidence interval above 0.05 were considered.

Patterns of admixture were also assessed using STRUCTURE (Pritchard et al. 2000). We assumed that each species should be assigned to at least one independent cluster and individuals showing mixed assignments are indicative of admixture. This analysis was done with the codominant matrix obtained from the genotyping pipeline. STRUCTURE ran for K values between 1 and 23 (the maximum number of species) for 100 K generations after a burn‐in of 100 K, with 10 iterations each. The best K value was tested using the DeltaK as implemented in Structure Harvester (Earl and VonHoldt 2012).

### 2.5 cpDNA

A chloroplast DNA matrix was produced in the context of a parallel work (Puppo et al. in prep.) for 5540 nucleotides and 473 individuals covering all species from the Canary Islands. These included 12 amplicon markers genotyped with the same approach described for the nuclear markers. All samples genotyped with EPIC markers were also included in this dataset. To have a direct comparison between variation portrayed by nuclear and plastid genomes, the data from the individuals in common with the nuclear dataset was extracted. This dataset was analyzed the same way as the nuclear dataset with the following differences: as described in Puppo et al. (in prep), genotype calling considered haploid loci and therefore the most likely variant was considered; indels and an inversion were coded for the phylogenetic analysis to gain additional information; phylogenetic analysis with MrBayes ran for 200 M generations, and the burn‐in was set to 100 M; haplotype sharing values were calculated taking the concatenated matrix and not per marker. Additionally, a haplotype network was produced with the program PopArt (http://popart.otago.ac.nz). To this end, samples containing missing data for any of the markers were excluded.

## 3 Results

### 3.1 Nuclear Markers Development and Genotyping Success

The genomes of four *Micromeria* individuals were assembled to serve as resources for marker development. All data, including short reads and assembly sequences, are available at NCBI under the Bioproject PRJNA1193890. Between 26.7 and 32.2 M paired reads were produced per sample; between 99.7% and 99.8% passed the quality control (Table S1). Assemblies consisted of between 50,874 and 77,232 scaffolds above 200 bp, summing up to between 138 Mbp and 146 Mbp. N50 varied between 4461 bp and 7547 bp. Between 82% and 95% BUSCOs were found depending on the assembly and database (Table S1).

Of the 24 primers designed, only one (IPK) did not show any amplification in the first in vitro PCR test (Table S3). The resulting markers amplified at least three of the four *Micromeria* samples in the initial test and were employed in the genotyping of the full sampling set. A total of 5.6 M paired reads were produced in the Illumina run (average of 19.8 K per sample) from which 5.3 M paired reads passed the quality control step and were used for the genotyping process. After filtering samples and markers based on missing data, 14 EPIC nuclear markers and 231 samples were kept and used for further analyses (see Table S2). Both nuclear and cpDNA short sequence data are available NCBI under the Bioproject PRJNA1194140.

### 3.2 Phylogenetic Patterns

**FIGURE 1:**
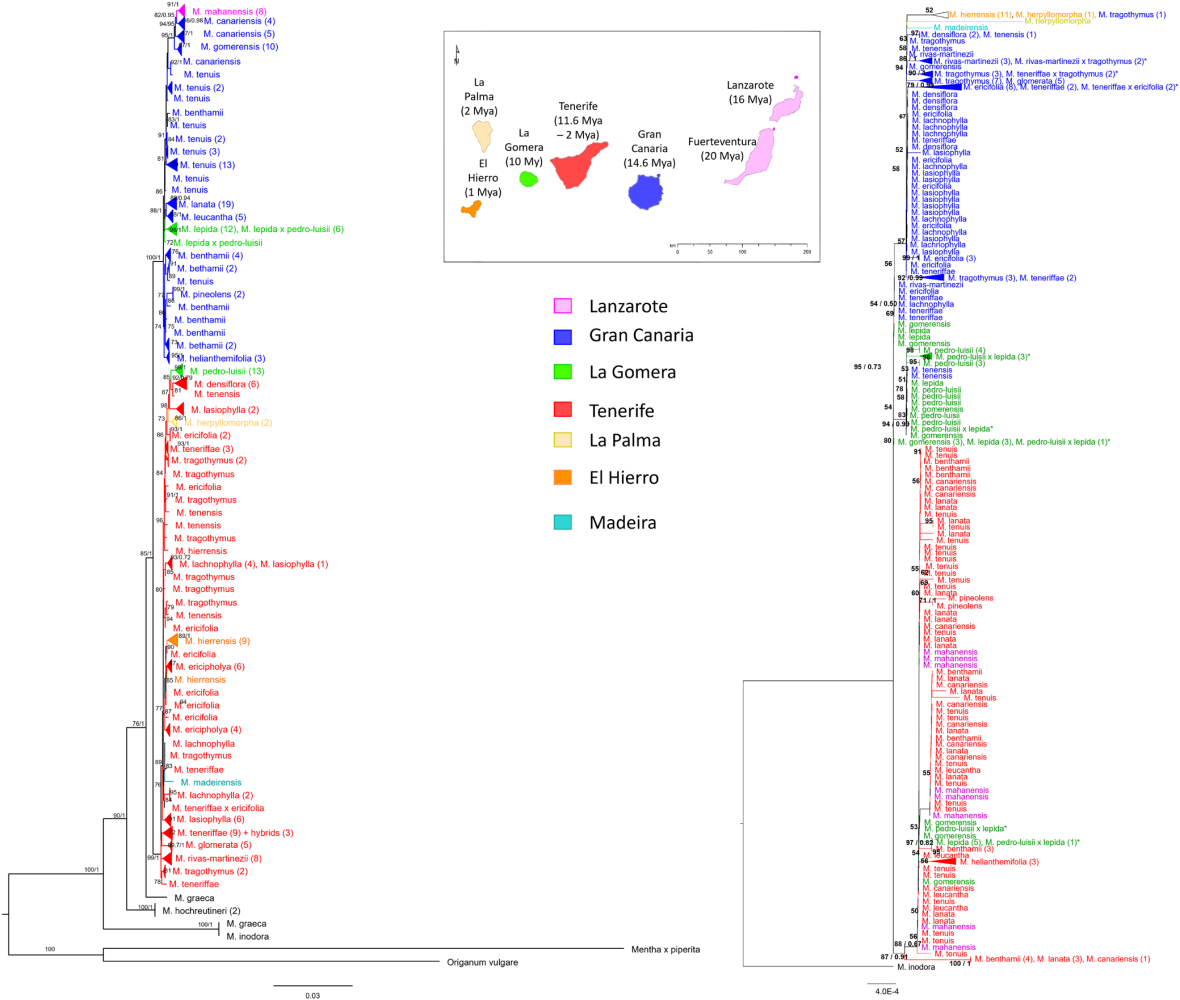
Phylogenetic tree estimated with the concatenated dataset of the 14 nuclear markers (left) and cpDNA (right) using IQ‐Tree. Branch support values correspond to bootstrap values (left) posterior probabilities of clades in common with Mr. Bayes analyses (right). Only bootstrap values and posterior probabilities above 50% and 0.5, respectively, are shown. Clades and taxon names are colored based on island of origin. Some groups composed of more than three individuals are collapsed to facilitate visualization. In these cases, the number of samples are written in parenthesis next to the taxa names. Furthermore, to facilitate visualization morphological hybrid individuals were marked with an asterisk.

Overall, the major phylogenetic patterns portrayed by the nuclear markers are congruent with previous studies (Figures 1 and S1). In both maximum likelihood and Bayesian analyses, samples are divided into two main lineages previously described for *Micromeria* (Meimberg et al. 2006; Puppo et al. 2014, 2015), East (Lanzarote, Gran Canaria and *M. lepida* and *M. gomerensis* of La Gomera) and West (Tenerife, La Palma, El Hierro, Madeira and *M. pedro‐luisii* from La Gomera). Within each lineage, taxa from Gran Canaria and Tenerife are paraphyletic with species from other islands. Within the eastern group, three clades are formed: (1) *M. canariensis* (P.Pérez) Puppo (Gran Canaria), *M. mahanensis* (Lanzarote) and *M. gomerensis* (La Gomera) (PP = 1, bootstrap = 77%); (2) *M. lanata* Benth. (Gran Canaria), *M. leucantha* Svent. ex P. Pérez (Gran Canaria), *M. lepida* , and hybrids involving this species (La Gomera) (PP = 1); (3) *M. helianthemifolia* Webb & Berthel., most of the *M. benthamii* Webb & Berthel. samples, and *M. pineolens* Svent. (all from Gran Canaria, PP = 0.8). *Micromeria tenuis* (Link) Benth. (Gran Canaria) appeared paraphyletic to clade 1 with low resolution and support values (PP = 0.6). Overall, the western lineage had a lack of resolution in the Bayesian analysis when it comes to phylogenetic relationships, and most of it formed a large polytomy. In the maximum likelihood tree, some major clades were formed within the western lineage but all with low support values. For most species, there was a clade where most of the individuals were included. However, some species were interspersed with samples from other species. This was the case for *M. tragothymus* Webb & Berthel., *M. tenensis*, and *M. ericifolia* within the western lineage and *M. benthamii* and *M. lanata* within the eastern lineage. Times of divergence were in the range from those obtained by Puppo et al. (2014) (Figure S2). The split between the main lineages was 7.4 Mya, and the diversification within each lineage started 5.9 Mya for the west and 7.1 Mya for the east. Overall timings of divergence among species were lower in the western lineage than in the eastern. For example, the lowest sptime of divergence in the eastern lineage was 5.0 Mya (between *M. canariensis* and *M. mahanensis*), while in the west, it was 1.2 Mya (between *M. ericifolia* and *M. hierrensis*).

### 3.3 cpDNA Haplotype Structure and Phylogeny

**FIGURE 2:**
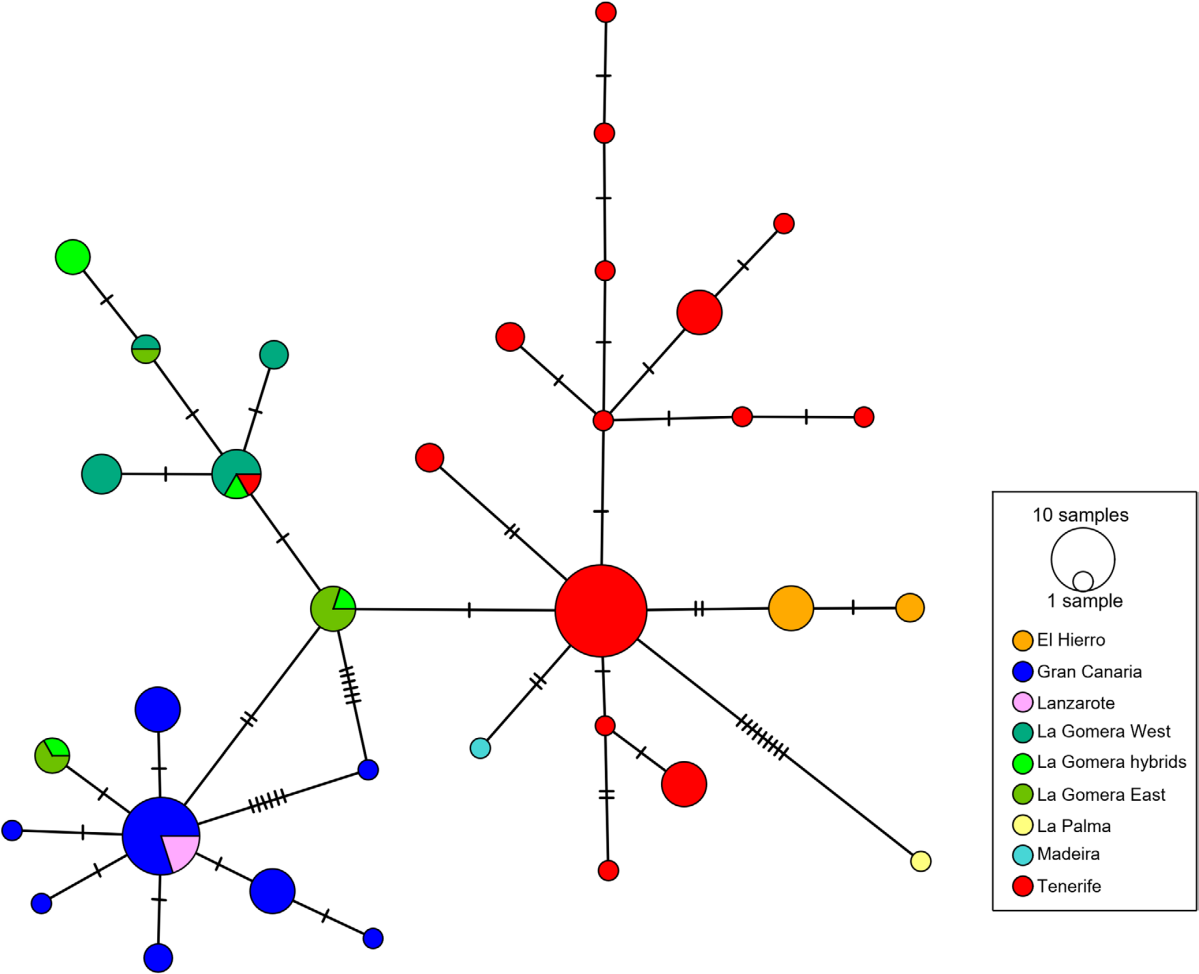
Haplotype network using 4063 bp of the chloroplast genome across 275 samples. Each pie chart corresponds to a haplotype that is color coded based on the island of origin. La Gomera east and west lineages and corresponding hybrids are coded with different colors. Pie chart size is proportional to number of samples sharing that haplotype. Each dash on the connection edges between haplotypes corresponds to a nucleotide substitution.

The haplotype network of the chloroplast data included 107 individuals and showed little divergence between haplotypes being most of them shared between species and many between islands (Figure 2). Nevertheless, a pattern of structure between haplotypes is observed, making it possible to outline three main groups. These reflect a separation between the eastern and western lineages, with the difference that most haplotypes from La Gomera form a third intermediate group. Interestingly, two haplotypes are shared between the eastern and western lineages in samples from *M. lepida* , *M. pedro‐luisii*, and morphological hybrids from La Gomera, and with *M. tenensis* in Tenerife. Phylogenetic analysis resulted in three main clades (Figures 1 and S1): (1) includes most of the western lineage (excluding La Gomera); (2) corresponds to the eastern lineage; and (3) includes individuals from the three species found in La Gomera and two individuals from *M. tenensis* (Tenerife). Morphological hybrids between *M. lepida* and *M. pedro‐luisii* are found both in the second and the third clades. There was little resolution within each of these clades, and most species were polyphyletic.

### 3.4 Genetic Diversity and Differentiation

**FIGURE 3:**
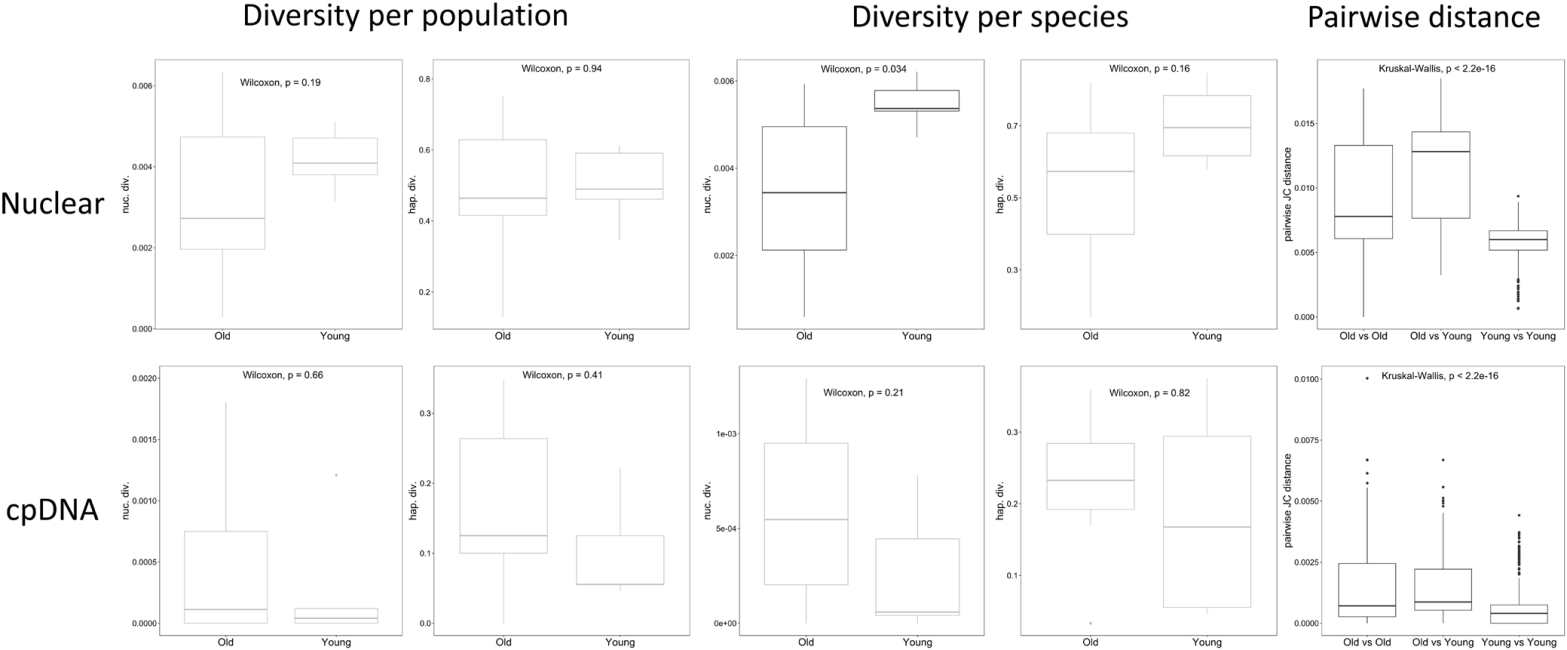
Genetic diversity and sequence divergence comparison between older and younger islands for the EPIC nuclear (top) and cpDNA (bottom) marker sets. Genetic diversity was estimated based on nucleotide and haplotype diversity calculated per population (left) and species (middle). Sequence divergence was tested based on pairwise sequence distance among all individuals (right). Boxplots of significant tests are in black while non‐significant in gray.

**FIGURE 4:**
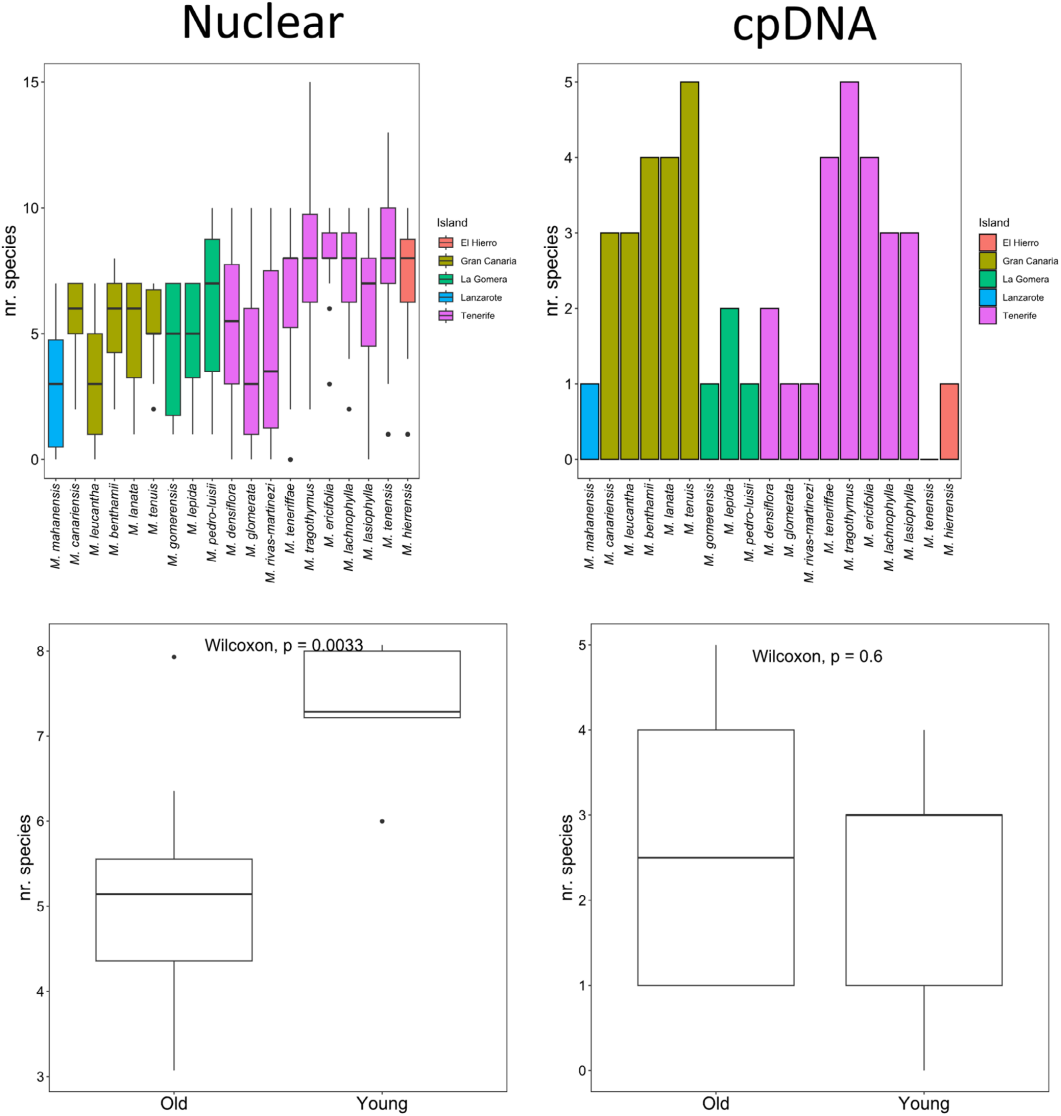
Number of species sharing haplotypes with each of the 17 *Micromeria* taxa represented by at least five samples. The left plots summarize the number of haplotypes shared per taxon (top) and by old vs. young taxa (bottom) across the 14 EPIC nuclear markers, while the ones on the right show the results for the cpDNA dataset.

Genetic diversity was estimated based on nucleotide and haplotype diversity from populations and species groupings. Nucleotide and haplotype diversity values for the nuclear markers and the chloroplast data per population and species are listed in Table S4. Mean nucleotide diversity across the 14 EPIC markers was significantly higher in younger taxa than in older ones (Figure 3). Although not significant, the same trend was found for comparisons at the population level and using haplotype diversity. This pattern was not equally supported by all loci. Five loci for population and species groupings showed higher nucleotide diversity for younger taxa (Figure S3). Only two markers showed significantly higher haplotype diversity (both at species and population level) in younger islands than in older ones (Figure S4). Although the same trend of higher diversity in younger than in older islands was observed for the cpDNA dataset, this difference was not significant (Figure 4). As expected, genetic differentiation patterns showed the opposite trend, being higher among older than younger islands for both nuclear and cpDNA (Figure 4). Pairwise distance between *old and young* species was generally higher than the other comparisons. Although all comparisons showed significant differences, this pattern was not obvious for all markers (Figure S5).

### 3.5 Haplotype Sharing Patterns

Overall, there was a significant difference between younger and older islands with regard to the number of species with which each taxon shares haplotypes (Figure 4). For the nuclear markers, *M. ericifolia* (Tenerife, younger area) was the taxon that shared haplotypes with the most species, an average of 8.07 species per marker, while *M. mahanensis* (Lanzarote and Fuerteventura) shared haplotypes with the least number of taxa (3.07). This pattern is similar to the one observed with genetic diversity, where species growing on younger islands shared haplotypes with a higher number of species than those growing on older islands (Figure 4). This pattern was supported by nine of the 14 markers (Figure S6). *Micromeria tenensis* (Tenerife, older area) was the only species that did not share chloroplast haplotypes with other taxa, while *M. tenuis* (Gran Canaria) and *M. tragothymus* (Tenerife, younger area) shared haplotypes with most species (Table S4). There was no significant difference between older and younger species in haplotype sharing patterns for chloroplast markers.

**FIGURE 5:**
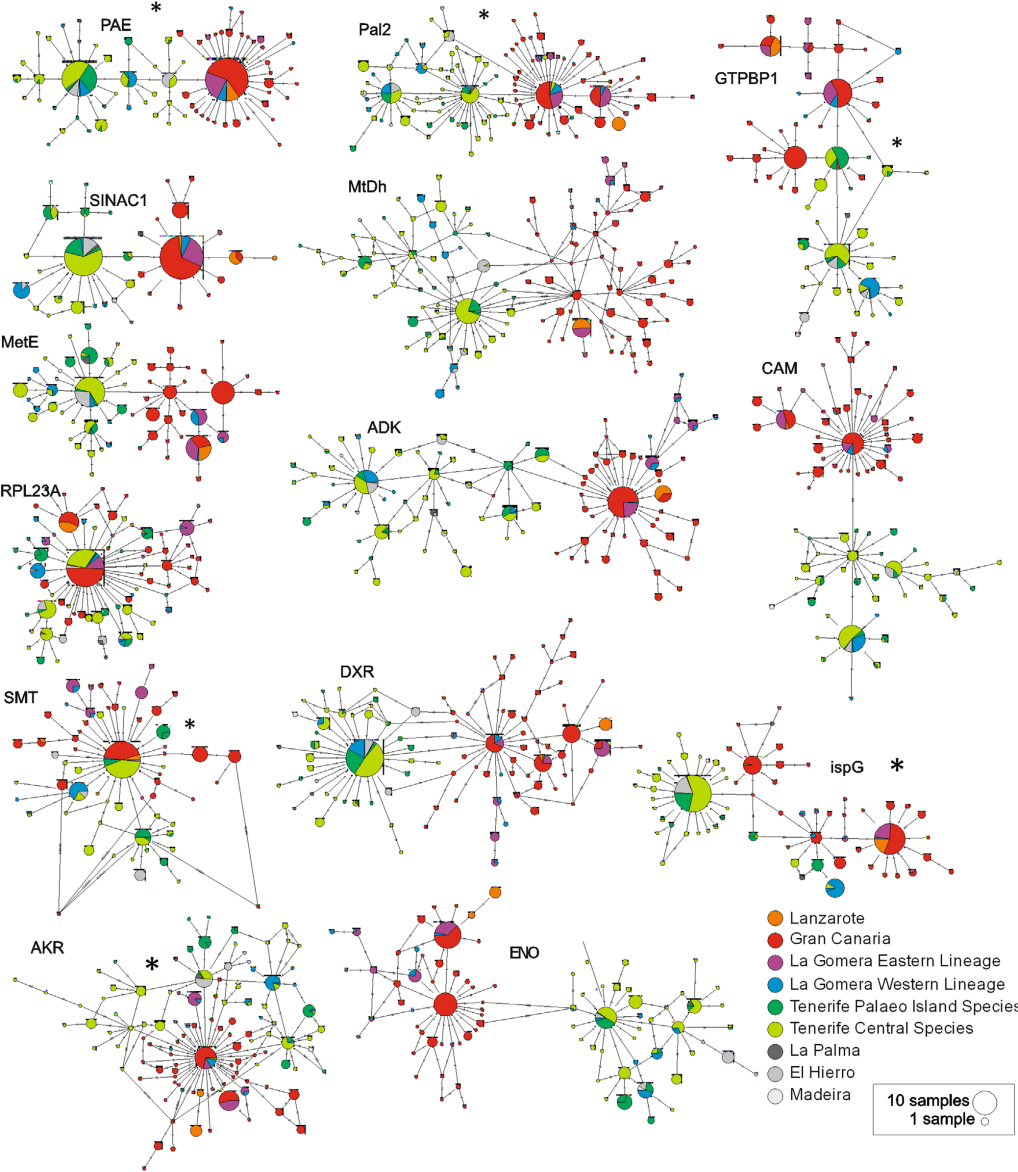
Haplotype networks for each EPIC marker. Each pie chart corresponds to a haplotype that is color coded based on the island of origin. La Gomera east and west lineages and corresponding hybrids, as well as Tenerife's paleo‐island and central species, are coded with different colors. Pie chart size is proportional to number of samples sharing that haplotype. Each dash on the connection edges between haplotypes corresponds to a nucleotide substitution. Networks marked with asterisk (*) show haplotype sharing between the east and west lineages.

Haplotype sharing patterns were further evaluated through haplotype networks (Figure 5). A haplotype structure congruent with the East–West split was found for 10 nuclear markers. However, for three of them, there was at least one haplotype shared between the species of these two groups. In most cases, eastern haplotypes were detected in species from the western lineage, for example, *M. tragothymus* (Tenerife, younger area), *M. tenensis* (Tenerife, older area), and *M. herpyllomorpha* (La Palma). For one marker (ADK), there was one haplotype only found in *M. tenuis* (Gran Canaria, eastern lineage) that grouped together with the western lineage. From the remaining markers, the haplotype network of GTPBP1 and ispG showed some structure that was not sorted according to the East–West lineages, and RPL23A and SMT showed no structure at all, with the major haplotypes shared across both lineages.

### 3.6 Interspecific Gene Flow

**FIGURE 6:**
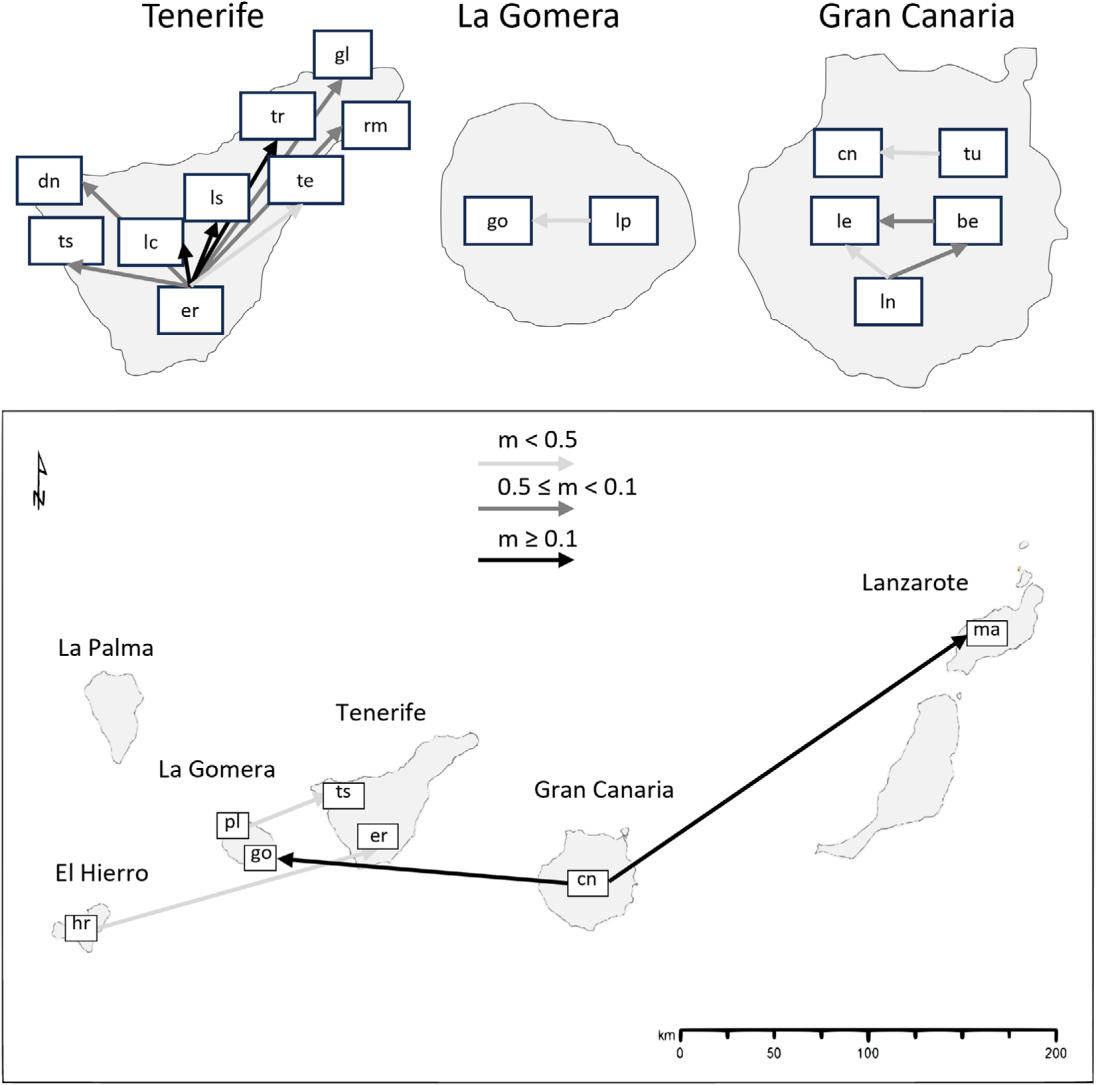
Representation of significant recent migration rates, estimated with BayesASS, between species on the same island (top) and between islands (bottom). Arrows reflect the direction of the migration rate. Darker colored arrows represent higher amounts of migration rates. Species initials are as follow: be—*M. benthamii*, cn— *M. canariensis* , dn— *M. densiflora* , er— *M. ericifolia* , gl— *M. glomerata* , go—*M. gomerensis*, le—*M. leucantha*, hr.—*M. hierrensis*, ln— *M. lanata* , lc—*M. lachnophylla*, ls— *M. lasiophylla* , ma—*M. mahanensis*, pl.—*M. pedro‐luisii*, rm.—*M. rivas‐martinezii*, ts—*M. tenensis*, te—*M. teneriffae*, tr—*M. tragothymus*, tu— *M. tenuis* .

Significant recent migration rates between species calculated with BayesASS varied between 0.02 ( *M. lanata* to *M. leucantha* ) and 0.15 ( *M. ericifolia* to *M. tragothymus*; Table S5). Most of them were found within islands. Tenerife was the island that showed the highest number of connections (8) followed by Gran Canaria (4) and La Gomera (1) (Figure 6, Table S5). Tenerife also showed the highest values of migration rates, all of them originating from *M. ericifolia* and targeting all remaining species from this island. In Gran Canaria, significant migration rates were found in two groups of species: (1) from *M. tenuis* to *M. canariensis* and (2) from *M. lanata* to *M. benthamii* and *M. leucantha* , and from *M. benthamii* to *M. leucantha* . In La Gomera, a significant migration rate was found from *M. lepida* to *M. gomerensis*. Evidence of inter‐island gene flow was found from *M. canariensis* (Gran Canaria) to *M. mahanensis* (Lanzarote) and *M. gomerensis* (La Gomera); from *M. hierrensis* (El Hierro) to *M. ericifolia* (Tenerife); and from *M. pedro‐luisii* (Tenerife) to *M. tenensis* (Tenerife).

### 3.7 Genetic Structure

**FIGURE 7:**
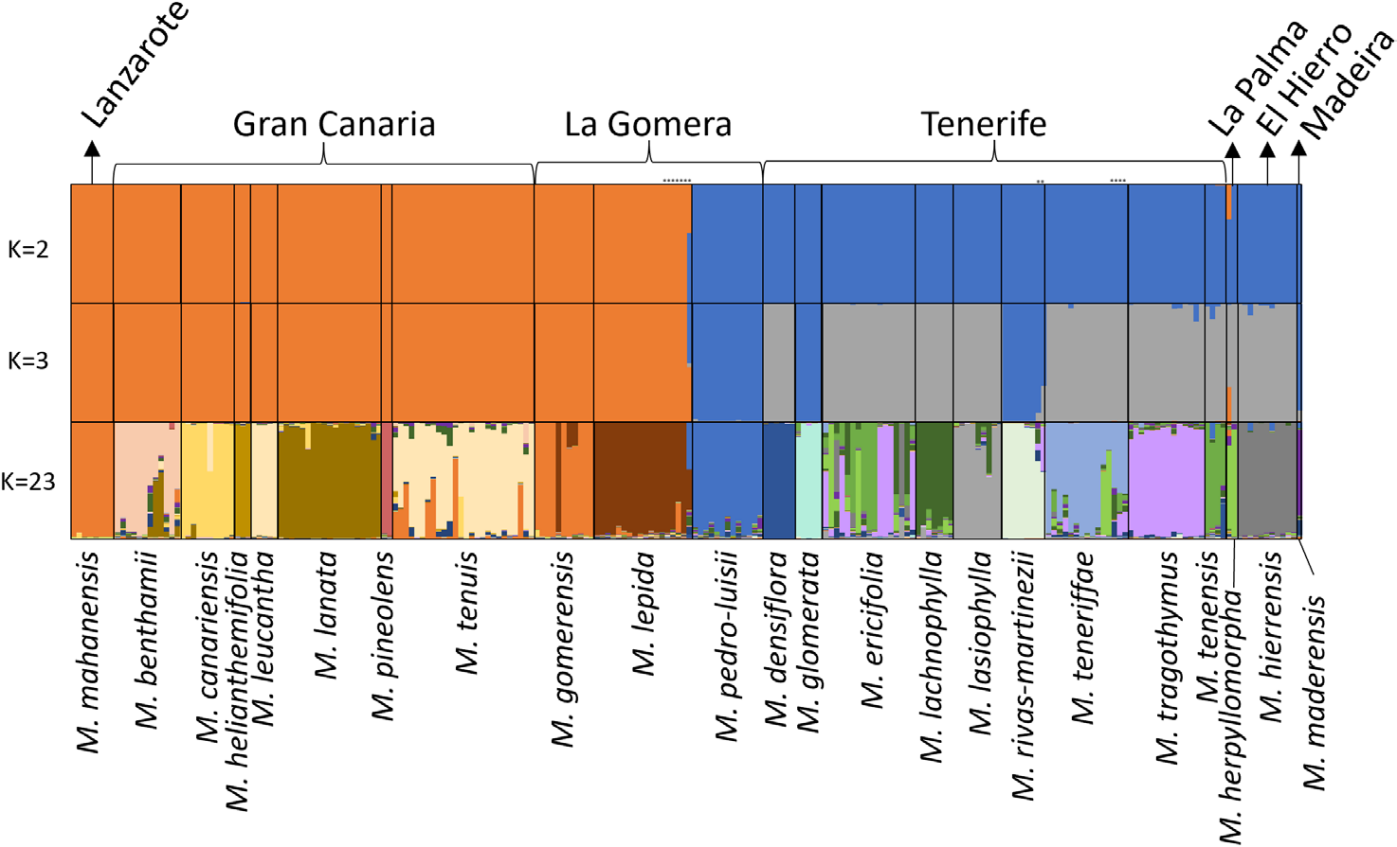
STRUCTURE analyses of the species of *Micromeria* in the Canary Islands showing selected K values. Optimum K according to the DeltaK method is K = 3.

The optimum K value according to the DeltaK method was 3 (Figure S7). Results for two aditional K values are shown (Figure 7): K = 2, since *Micromeria* variation is organized into the two main lineages described in the phylogenetic analysis, east and west; and K = 23, since it is the number of species included in this study. As expected, cluster assignment for K = 2 split samples into the east and west lineages. Only one sample, a morphological hybrid individual between *M. lepida* and *M. pedro‐luisii* from La Gomera, shows a mixed assignment to both clusters. For K = 3, the western lineage is further divided into two groups: one containing *M. pedro‐luisii* (La Gomera), *M. glomerata* , and *M. rivas‐martinezii* (Tenerife, older areas), and *M. maderensis* (Madeira), and a second group including all remaining taxa. For K = 23, most species form their own cluster. Exceptions are the species pairs *M. mahanensis* (Lanzarote) and *M. gomerensis* (La Gomera), and *M. leucantha* and *M. tenuis* (both from Gran Canaria). The most widespread species in Tenerife, *M. ericifolia* , is assigned to multiple clusters, showing a high degree of admixture with other species. Most individuals of *M. ericifolia* shared clusters with two other species of Tenerife, *M. tragothymus* and *M. tenensis*. Admixed individuals were observed for other species, being more prevalent in other widespread species like *M. benthamii* and *M. tenuis* in Gran Canaria. Several morphological hybrid individuals were included in this analysis. As mentioned above, only one out of the seven morphological hybrids between *M. lepida* and *M. pedro‐luisii* was assigned to clusters from both species. The morphological hybrids involving the remaining species showed mixed assignment according to the prediction: two *M. rivas‐martinezii* × *M. tragothymus* individuals, two *M. teneriffae* × *M. ericifolia* individuals, and two *M. teneriffae* × *M. tragothymus* individuals.

## 4 Discussion

### 4.1 Syngameon Dynamics in *Micromeria*

Patterns of genetic diversity and structure of *Micromeria* in the Canary Islands allowed us to infer multiple factors for the formation, expansion, and maintenance of a syngameon. The interplay of island ontogeny, evolutionary divergence, range size, and inter‐island gene flow results in a connectivity between the species that is within the expectation of the surfing syngameon hypothesis (SSH; Caujapé‐Castells et al. 2017). Genetic structure and phylogenetic patterns correspond generally to the results obtained in previous studies such as the split into two main lineages (Curto et al. 2017, 2018; Meimberg et al. 2006; Puppo et al. 2014, 2015, 2016). In addition, the higher genetic diversity of species growing on younger islands is congruent with earlier studies using microsatellite loci (Curto et al. 2017). The use of GBAS allowed us to combine sequence and frequency information, providing simultaneously a phylogenetic and population genetics perspective to the syngameon dynamics. Furthermore, GBAS generates phased sequence information, opening the possibility to evaluate patterns of haplotype sharing, sequence divergence, and nucleotide diversity. These provide extra levels of information. The evaluation of haplotype sharing patterns provided information about the role that different species may play in the syngameons. Sequence divergence allowed us to evaluate differentiation patterns at the individual level, overcoming limitations of unequal sampling across populations. Finally, nucleotide diversity was highly informative in retrieving genetic diversity patterns. Contrary to frequency‐based measures like haplotype diversity, nucleotide diversity is based on sequence divergence (Hedrick 2009). This makes nucleotide diversity more efficient in retrieving genetic diversity patterns in populations represented by low sampling compared to haplotype diversity (Goodall‐Copestake et al. 2012). Moreover, nucleotide diversity can be inflated when divergent alleles are integrated into a species' gene pool through introgression (Sweigart and Willis 2003). This makes GBAS especially suitable to investigate the role of syngameon dynamics in sharing diversity across species borders. The stronger increase of nucleotide diversity compared to haplotype diversity on younger versus older parts of the archipelago underlines the role of interspecific gene flow in explaining these patterns.

Our results also show that species in the younger parts of the archipelago share haplotypes with more taxa than species on older islands. Additionally, widespread taxa seem to share haplotypes with more species than taxa with narrow distributions. Shared haplotypes can be explained by markers conserved across recently diverged lineages, by incomplete lineage sorting (ILS) and introgression (López‐Alvarado et al. 2014). Previously, phylogenetic analyses showed the existence of recently diverged species (e.g., *M. mahanensis* from Lanzarote) on both younger and older islands (Puppo et al. 2015). If the haplotype sharing patterns were mainly explained by a lack of marker differentiation or ILS, haplotypes should be shared between recently diverged lineages on all islands and not predominantly on younger islands. Haplotype networks confirm that western species, which are generally younger, share haplotypes with the eastern species at low frequencies but not the other way around. Furthermore, the intraspecific migration rates estimated with BayesASS reveal similar patterns, with younger lineages being connected by gene flow. While these estimates could be inflated due to limited marker resolution, this is unlikely, as STRUCTURE analysis shows that most species form distinct clusters, indicating sufficient genetic variation to differentiate them. Notably, the admixture patterns observed in STRUCTURE align with both haplotype sharing and migration rate estimates. The consistency across multiple independent methods, each using different types of information, strongly supports that introgression is the most likely explanation. Based on these results, species syngameons are more interconnected in the western, younger islands than in older ones, where it is more fragmented. The SSH postulates that island geomorphology is the main motor shaping syngameons in oceanic islands (Caujapé‐Castells et al. 2017). On the contrary, the formation of new land and increase in altitudinal range promote the expansion and formation of new syngameons by increasing the island's ecological opportunities for species differentiation, while factors like erosion counteract this and promote stochastic processes of speciation and loss of genetic variation. This is illustrated by the contrast between the island of Lanzarote (15.5 Mya) and El Hierro (1.1 Mya). Both contain single island endemics, *M. hierrensis* (El Hierro) and *M. mahanensis* (Lanzarote), that are thought to have diverged recently from species in Tenerife and Gran Canaria, respectively (Puppo et al. 2015). However, *M. hierrensis* shows a much higher genetic diversity and shares haplotypes with more species than *M. mahanensis*. El Hierro is still increasing its topographic complexity due to relatively frequent volcanic activity, while Lanzarote has a less pronounced height profile, and erosion is the main geological force shaping the island (Carracedo 2011). The higher ecological complexity of El Hierro might have promoted the establishment of multiple colonizing lineages, while in Lanzarote, niche preemption could have prevented the arrival of new genetic variation to the island.

Volcanic activity has been shown to contribute to the expansion of syngameons within the same island. The most prominent example is Tenerife, where the central part of the island was formed by the emergence of a recent volcano, the Teide (Ancochea et al. 1990), that connected three paleo‐islands, each of different geological ages. Thus, Tenerife in its current shape is composed of three old areas, remnants of the original paleo‐islands, and a central, recent substrate. This resulted in the formation of a complex syngameon in Tenerife involving all species of *Micromeria*, as shown by the significant interspecific migration rates between all nine species present on the island. Moreover, individuals from taxa growing in the central, younger part of the island show high genetic similarity to other species in both phylogenetic and structural analyses. To a lesser extent, this has also been the case with Gran Canaria, although Gran Canaria was not formed due to secondary connections of previously isolated areas like Tenerife. Rather, volcanic activity occurred at different stages in different parts of the island. The SW half of Gran Canaria is composed of Miocene substrates, while the NE half was revitalized by recent volcanic eruptions in the Pliocene (del‐Arco et al. 2002). The geological history of Gran Canaria did not have the same impact in increasing *Micromeria's* syngameon as it had in Tenerife. The newly formed part of Gran Canaria was contiguous to the existing part, and it occupied a lower portion of the island than Tenerife. The colonization of this new part likely occurred through range expansion of the existing *Micromeria* species. Although they likely formed new contact zones and interspecific gene flow because they remained in contact with already existing populations, its impact in the form of homogenizing diversity and breaking species' boundaries was lower than in Tenerife. Only five out of seven *Micromeria* species in Gran Canaria show evidence of interspecific migration rates, and genetic delimitation of species boundaries is clearer than in Tenerife. In Tenerife, the younger part of the island corresponds to most of its area, and it is likely that at some point it was detached from the older parts by landslides or other processes (Ancochea et al. 1990). Thus, its colonization happened with isolation from the colonization source, and therefore the new syngameons had a higher impact in homogenizing genetic diversity across species boundaries. The allopatric pattern of species' ranges in central Tenerife underlines the possibility of a higher exchange rate and a more dynamic syngameon at the contact zones. In Gran Canaria, barriers to gene flow might already be more developed.

Intrinsic barriers to gene flow play an important role in species syngameon processes. In the island of La Gomera, species belonging to the Eastern and Western lineages can form morphological hybrid forms. For example, morphological hybrids between *M. lepida* (East) and *M. gomerensis* (West) are found in La Gomera; however, only one of them was confirmed with the STRUCTURE results, and no admixture between these species was supported by the current nuclear markers and microsatellite work from Curto et al. (2017). So, despite the formation of morphological hybrids, no sign of recent introgression was found between these species, indicating strong postzygotic barriers. These species are estimated to have diverged with the diversification of *Micromeria* in the archipelago (~8 Mya as in Puppo et al. 2023; and 7 Mya in the current work), increasing the likelihood for the accumulation of strong postzygotic barriers to gene flow. Contrary to this, all morphological hybrids involving other species showed the expected admixture pattern, most involving species that diverged more recently (maximum of 6 Mya), suggesting barriers to gene flow may not be as strong. These results support the idea that after a certain degree of divergence, species become disconnected from the syngameon. The difference between the syngameons in Tenerife and Gran Canaria could reflect this. In Gran Canaria, most of the species are organized in lineages whose divergence is older (7 to 4 Mya) than Tenerife species (6 to 1 Mya). As already reported in Puppo et al. (2014) the most recent divergent times in Tenerife coincide with the emergence of the central part of the island. In this case, the probability of a syngameon establishing and expanding would be a function of volcanic activity and syngameons age. A high degree of gene flow supported by early stages of the syngameons causes a higher similarity between species in the younger island parts. Conversely, with the age of the syngameon and the more specific adaptations needed at a certain locality, the postzygotic isolation mechanisms arise, and the species become increasingly disconnected from the syngameon. This might also explain the lower number of shared haplotypes in the paleo‐island species in Tenerife. Because of this dynamic, highly connected syngameons are present during colonization, whereas more detached ones appear at the rear end, where species are more likely to be completely isolated from any syngameon.

### 4.2 The Role of Widespread Species in Maintaining the Syngameon

In line with these arguments is the finding that widespread species seem to provide more diversity to the syngameon than the more narrowly distributed taxa. The most widespread species on the island of Tenerife, *M. ericifolia* (formerly *M. hyssopifolia*), grows across most of the island's area and biomes (Puppo et al. 2014, 2016). This species is a source of gene flow indicated by a significant migration rate to all remaining species and shows the highest levels of admixture and phylogenetic affinity to other taxa. To a lower extent, similar patterns were verified for other widespread species such as *M. benthamii* and *M. tenuis* from Gran Canaria. Taxa with the ability to form a higher number of hybrid connections than the remaining taxa have been described as hubs of introgression (Buck and Flores‐Rentería 2022). These are found in both mainland and island syngameons like the American southwestern pinyon pine (Buck et al. 2023) and genus *Parolinia* from Gran Canaria (González‐Pérez and Caujapé‐Castells 2022). Concerning the latter, González‐Pérez and Caujapé‐Castells (2022) hypothesized that recent volcanic activity in Gran Canaria promoted the expansion of some species, allowing the establishment of secondary contact and new hybrid connections. The distribution pattern of *M. ericifolia* supports this, as it grows in most younger parts of Tenerife and might have expanded from the paleo‐island region of Roque del Conde, where it is the only species occurring today. However, our data show that other processes need to be considered as well. *Micromeria ericifolia* shows a highly admixed gene pool indicating that it might have received substantial gene flow from other species during the expansion process. Introgression can promote range expansion through the incorporation of locally adapted variants from other species (Edelman and Mallet 2021), which would explain the ability of *M. ericifolia* to grow in such a wide range of ecological conditions. Signals of introgression correlated with adaptations to dry and wet conditions have been found in *M. canariensis* in Gran Canaria (Curto et al. 2018), so it is possible that similar processes explain the diversification of other *Micromeria* species in the archipelago. Thus, hubs of introgression might have been fueled by previous gene flow, which promoted taxa to have a wider distribution. The range size and wide ecological amplitude of *M. ericifolia* would thus be the result and the cause of the syngameon dynamics.

According to Buck and Flores‐Rentería (2022), these hubs of introgression might be important in maintaining the syngameons by slowing down differentiation after which hybrid connections can cease to exist and isolate species from the syngameon. They might also be important in the genomic mutualism mechanism proposed by Cannon and Lerdau (2015). When species share a part of their gene pool within a syngameon, they increase their effective population size. The authors hypothesize that this stabilizes rare species with a low effective population size by minimizing the effect of drift. Our results show connectivity between narrowly distributed (older) and widespread (younger) species indicating migration from younger to older taxa. This was the case of *M. densiflora, M. rivas‐martinezii*, and *M. glomerata* , narrow endemics from Tenerife, receiving migrants from the widespread *M. ericifolia* ; and *M. leucantha* from Gran Canaria receiving gene flow from the more ubiquitous *M. benthamii*. The narrow distribution of these species makes them more susceptible to stochastic events, but since they are also associated with a very narrow ecological range, competition with other taxa is reduced. Under the genomic mutualism scenario, introgression from the more widespread species may have helped maintain these species in the face of the extremely dynamic conditions of the islands. These narrow endemics constitute older lineages in the archipelago, are characterized by high morphological divergence, and are, in the case of Tenerife, restricted to the areas of the paleo‐islands (Puppo et al. 2014, 2015). Strong stabilizing selection has been proposed to be the main mechanism maintaining morphological integrity in syngameons (Stelkens and Seehausen 2009) and under the genomic mutualism scenario, would prevent the exchange of variants responsible for the divergent traits. The characteristics of the paleo‐island species would be retained in areas of extreme conditions by stabilizing selection of some parts of the genome. This would also imply that we recognize a group of populations as species only if they retain some morphological characteristics despite continuous gene flow within the syngameon. We do not know about the species that had been homogenized, even if they might have contributed pre‐adaptations to the syngameon's gene pool.

Related hypotheses can be tested through genome‐wide analyses by evaluating which variants can cross species borders and what their adaptive potential is (Cronk and Suarez‐Gonzalez 2018). These genomic studies are commonly focused on hybrid zone dynamics (Lewis et al. 2020) but not yet on species groups forming syngameons, and even less on oceanic islands (Cerca et al. 2023). We are currently working on closing this gap by sequencing the genome of multiple species of *Micromeria* from this archipelago. The incongruence in genetic diversity and haplotype sharing patterns found across different loci might be explained by the fact that the genome evolves at different rates due to different selection regimes (Senchina et al. 2003). Thus, different genomic regions have the potential to provide glimpses of the syngameon's dynamics across space and time.

### 4.3 Implications of Inter‐Island Gene Flow on Syngameons' Dynamics

Although we found that the syngameons are more connected within one island, we found that they can expand across several islands. We detected significant migration among species growing on different islands, indicating the existence of inter‐island gene flow. This involves species that are relatively closely related to each other and likely resulted from recent speciation events.

When analyzing haplotype structure, haplotypes are shared among the eastern and western lineages. More precisely, we see some individuals from the western, younger islands carrying haplotypes that are highly prevalent in the east. We observed this pattern in markers where there is a clear split between both lineages, so it is not caused by a lack of resolution. The most likely explanation is gene flow caused by colonization after the East/West split. Although the western part of the archipelago was mostly colonized by species from Tenerife, it is possible that it received some colonization from the eastern islands. The fact that we observed these haplotypes mostly in younger species can be related to their widespread and highly connected nature. They are at the center of the syngameons and therefore likely to receive variation from other taxa, including new colonizers. Also, their effective population size should be relatively larger than older, more narrowly distributed taxa, making the rare alleles resulting from interspecific gene flow less likely to be eliminated by drift. Thus, widespread species in younger parts of the archipelago may play an important role in receiving and maintaining variation from other islands.

Curto et al. (2018) observed a phylogenomic signal congruent with inter‐island gene flow and species ecology; concretely, that populations of *M. canariensis* growing in the laurel forest in Gran Canaria were more closely related to *M. gomerensis* from La Gomera, which grows mostly in this habitat, than to other *M. canariensis* populations. This suggests that syngameon dynamics may be important in bringing adaptive variation from one island to the other.

### 4.4 Concluding Remarks

Based on our results, we can postulate several hypotheses regarding the impacts of syngameons on the distribution of traits that impact the taxonomic outline of species. In a syngameon, interspecific gene flow homogenizes genetic variation, allowing for multiple lineages to merge. Nevertheless, species morphological integrity and distribution can somehow be maintained. In the case of *Micromeria*, many of these boundaries are likely defined by ecological conditions that stabilize certain variants that might be linked to morphological features used for species circumscription. Nevertheless, introgression within a syngameon may lead to morphological homogenization between ecologically differentiated populations, ecotypes, or species. In this case, different gene combinations would be stabilized by selection within the species at different localities. This explains the seemingly close phylogenetic relationship of species within one island despite their morphological divergence. In this way, the syngameon hypothesis allows for the enlargement of within‐species processes like range dynamics to species groups (Hampe and Petit 2005). This dynamic may nevertheless contribute to the sharing of some adaptive variation (Curto et al. 2018). In this context, syngameons might contribute to the widespread nature of some species since adaptive introgression may allow them to grow in very different conditions. With time, adaptation to narrow ecological conditions and speciation may contribute to the isolation of certain lineages, establishing postzygotic barriers and isolating them from the syngameon. This is an example of the species growing in the paleo‐island of Tenerife. Thus, the inclusion of new variants in these species might be rare.

We see that the most widespread species are the main contributors to the species syngameons, and therefore their differentiation may result in the syngameons becoming fragmented. Moreover, under a genomic mutualism scenario, they might play an important role in the resilience of taxa with lower effective population sizes, decreasing extinction rates. Conversely, these species might be this widespread because they benefited from adaptive introgression from other species, allowing them to occupy heterogeneous ecological conditions.

Most of these hypotheses will only be fully tested using whole genome sequencing data that contains both adaptive and non‐adaptive variation. As shown by our results, different parts of the genome may provide different perspectives about the syngameon dynamics, allowing us to have a more comprehensive picture of how it evolves in space and time. This includes the implementation of methods that leverage the high statistical power of genomic data to distinguish introgression from incomplete lineage sorting (ILS) among closely related species (e.g., D_FOIL_; Pease and Hahn 2015) and to infer the most likely demographic scenarios and parameters (e.g., fastsimcoal2; Excoffier et al. 2021). Despite the great development in the field of genomics in the last decades, the implementation of these methodologies on insular plant taxa are still sparse when compared to their mainland counterparts (Cerca et al. 2023). While this knowledge gap is not filled, many of the studies evaluating the impact of syngameons will remain highly speculative.

On a final note, the processes outlined in this study may be specific to some taxa from the Canary Islands and therefore might not reflect processes shaping diversity on other oceanic islands. A way to verify this would be through comparative analyses including multiple taxa from several archipelagos and ecological features. These would also allow expanding the range of factors that might affect the syngameons dynamic by including taxa with different evolutionary histories, ecological traits, and archipelagos with different characteristics. Commonalities among these would indicate the universality of the processes and therefore should be integrated into island biogeographical models or at least help in the development of general models explaining syngameon dynamics.

## Author Contributions

**Manuel Curto:** conceptualization (equal), data curation (lead), investigation (lead), methodology (lead), supervision (equal), validation (lead), writing – original draft (lead), writing – review and editing (equal). **Pamela Puppo:** conceptualization (supporting), investigation (supporting), validation (equal), visualization (equal), writing – review and editing (equal). **Harald Meimberg:** conceptualization (equal), formal analysis (equal), funding acquisition (lead), investigation (supporting), methodology (supporting), validation (equal), visualization (equal), writing – review and editing (equal).

## Conflicts of Interest

The authors declare no conflicts of interest.

## Supporting information


**Figure S1:** Phylogenetic trees estimated with the concatenated dataset of the 14 EPIC nuclear markers (left) and cpDNA (right) using Mr. Bayes. Branch support values correspond to posterior probabilities above 0.5. Clades and taxon names are colored based on island of origin. Some groups composed of more than three individuals were collapsed to facilitate visualization. In these cases, the number of samples are written in parenthesis next to the taxa names.
**Figure S2:**. Calibrate maximum likelihood tree using chronos function ape. Scale shows divergence times in million years from present.
**Figure S3:** Comparison of genetic diversity per population between older and younger islands estimated based on nucleotide (left) and haplotype (right) diversity. ns—non‐significant difference (*p* > 0.05), *significant difference (*p* < 0.05), **significant difference (*p* < 0.01).
**Figure S4:** Comparison of genetic diversity per species between older and younger islands estimated based on nucleotide (left) and haplotype (right) diversity. ns—non‐significant difference (*p* ≥ 0.05), *significant difference (*p* < 0.05), **significant difference (*p* < 0.01).
**Figure S5:** Comparison of genetic differentiation per species between older and younger islands estimated based on pairwise sequence divergence. ns—non‐significant difference (*p* ≥ 0.05), *significant difference (*p* < 0.05), **significant difference (*p* < 0.01).
**Figure S6:** Number of species sharing haplotypes for each of the included *Micromeria* taxa compared between older and younger islands for each marker. ns—non‐significant difference (*p* ≥ 0.05), *significant difference (*p* < 0.05), **significant difference (*p* < 0.01).
**Figure S7:** DeltaK and mean likelihood per K value estimated with Structure Harvester.

**Table S1:** Assembly statistics of four *Micromeria* genomes.
**Table S2:**. Information of samples used including species, population of origin, geographical coordinates, genotyping success, and sequencing throughput.
**Table S3:** Information of the EPIC markers used including gene of origin, primer sequence and characteristics, amplification, and genotyping success.
**Table S4:** Genetic diversity and haplotyping results per population and species for both EPIC makers and cpDNA.
**Table S5:** Migration rates calculated with BayesAss. Species 1 corresponds to the migration receiver and species 2 to the source. Ages 1 and 2 correspond to the age category assigned to the species 1 and 2, respectively.

## Data Availability

Raw sequencing data, and genome assemblies, are available under the NCBI's bioprojects PRJNA1193890 and PRJNA1194140. The data that support the findings of this study are openly available in the Supporting Information.
