# Supplementary material for: Nuclear and Chloroplast Markers Provide New Insights Into the Syngameon Dynamics of Genus Micromeria (Lamiaceae) in the Canary Islands
Source: Ecol Evol. 2025 Aug 2;15(8):e71843. doi: 10.1002/ece3.71843 (PMC12317368; doi:10.1002/ece3.71843)
Supplement: Supplementary file 1 — Figure S1: Phylogenetic trees estimated with the concatenated dataset of the 14 EPIC nuclear markers (left) and cpDNA (right) using Mr. Bayes. Branch support values correspond to posterior probabilities above 0.5. Clades and taxon names are colored based on island of origin. Some groups composed of more than three individuals were collapsed to facilitate visualization. In these cases, the number of samples are written in parenthesis next to the taxa names. Figure S2:. Calibrate maximum likelihood tree using chronos function ape. Scale shows divergence times in million years from present. Figure S3: Comparison of genetic diversity per population between older and younger islands estimated based on nucleotide (left) and haplotype (right) diversity. ns—non‐significant difference (p > 0.05), *significant difference (p < 0.05), **significant difference (p < 0.01). Figure S4: Comparison of genetic diversity per species between older and younger islands estimated based on nucleotide (left) and haplotype (right) diversity. ns—non‐significant difference (p ≥ 0.05), *significant difference (p < 0.05), **significant difference (p < 0.01). Figure S5: Comparison of genetic differentiation per species between older and younger islands estimated based on pairwise sequence divergence. ns—non‐significant difference (p ≥ 0.05), *significant difference (p < 0.05), **significant difference (p < 0.01). Figure S6: Number of species sharing haplotypes for each of the included Micromeria taxa compared between older and younger islands for each marker. ns—non‐significant difference (p ≥ 0.05), *significant difference (p < 0.05), **significant difference (p < 0.01). Figure S7: DeltaK and mean likelihood per K value estimated with Structure Harvester. [file ECE3-15-e71843-s002.docx]

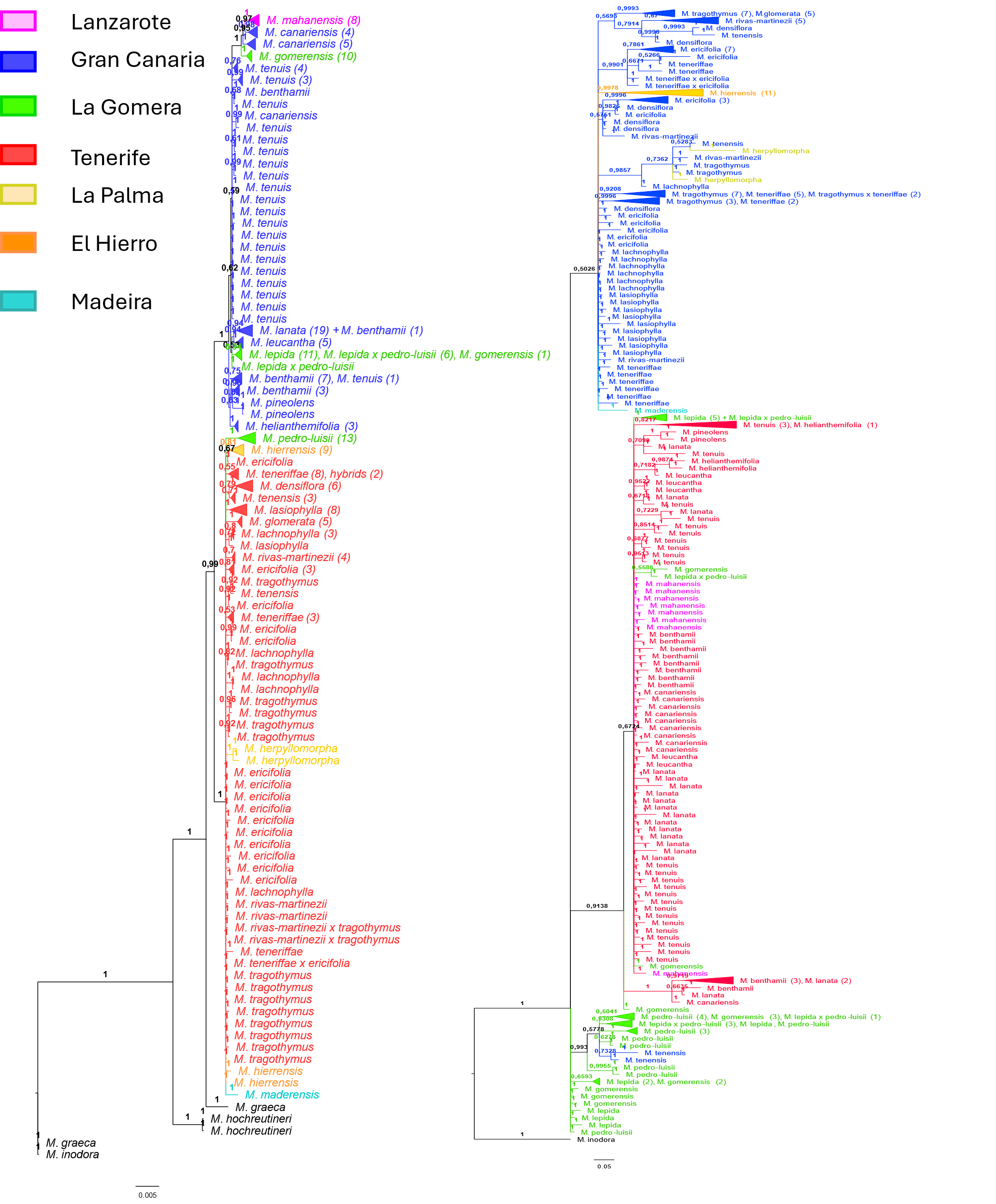


Figure S1. Phylogenetic trees estimated with the concatenated dataset of the 14 EPIC nuclear markers (left) and cpDNA (right) using Mr. Bayes. Branch support values correspond to posterior probabilities above 0.5. Clades and taxon names are colored based on island of origin. Some groups composed of more than three individuals were collapsed to facilitate visualization. In these cases, the number of samples are written in parenthesis next to the taxa names.


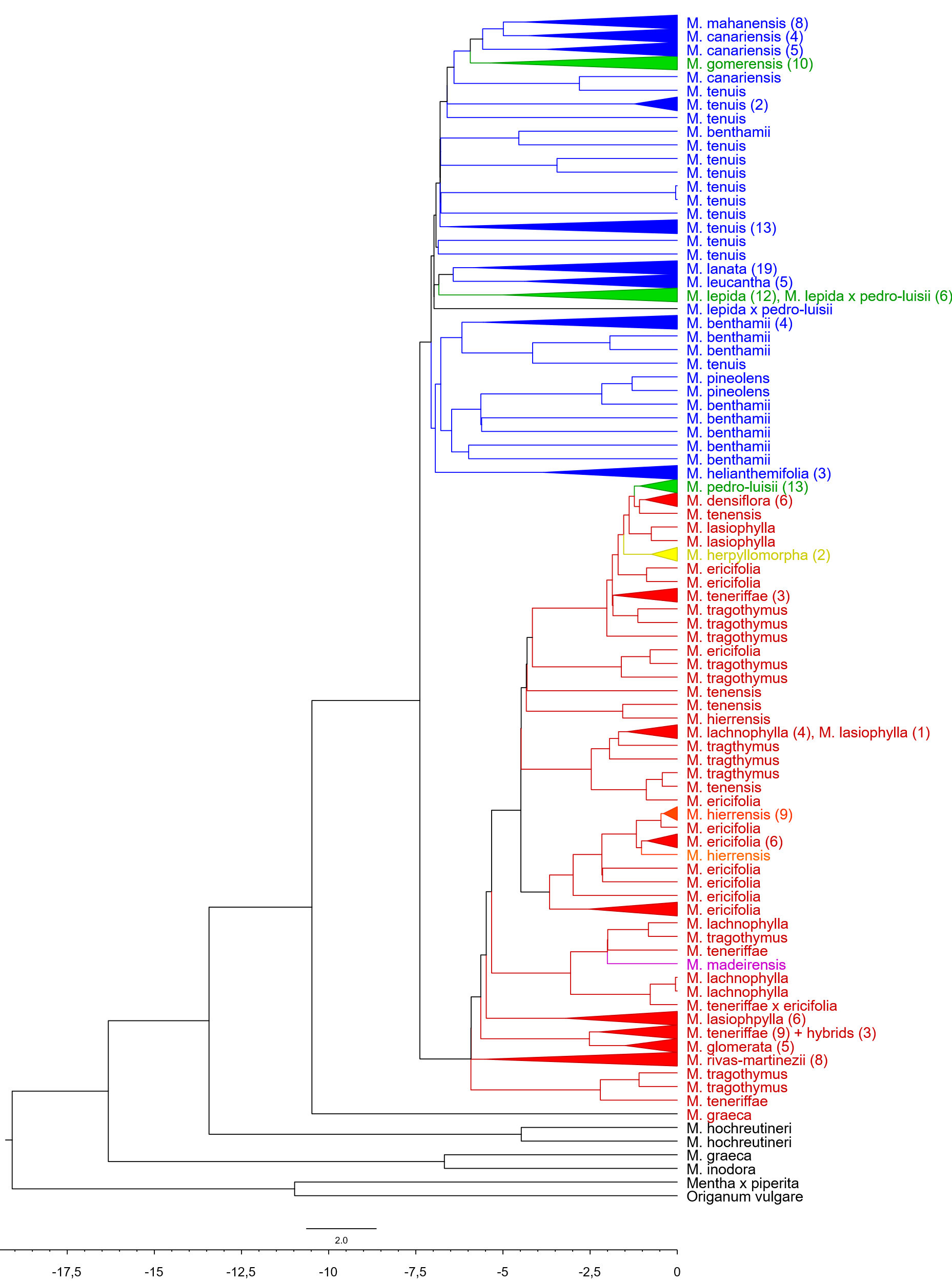


Figure S2. Calibrate maximum likelihood tree using chronos function ape. Scale shows divergence times in million years from present.

Supplementary Figure S3: Comparison of genetic diversity per population between older and younger islands estimated based on nucleotide (left) and haplotype (right) diversity. ns – nonsignificant difference (p > 0.05), * significant difference (p < 0.05), ** significant difference (p < 0.01).


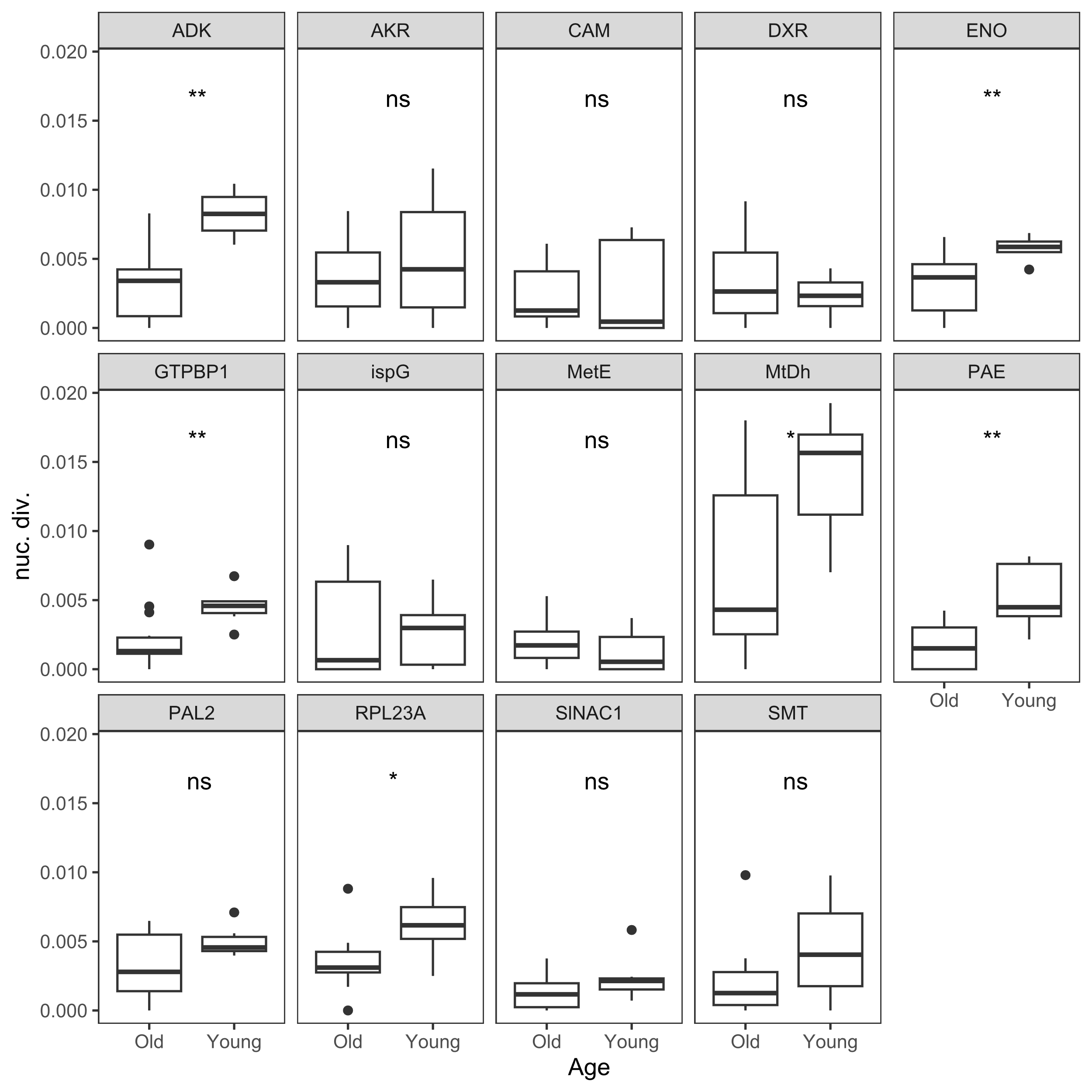

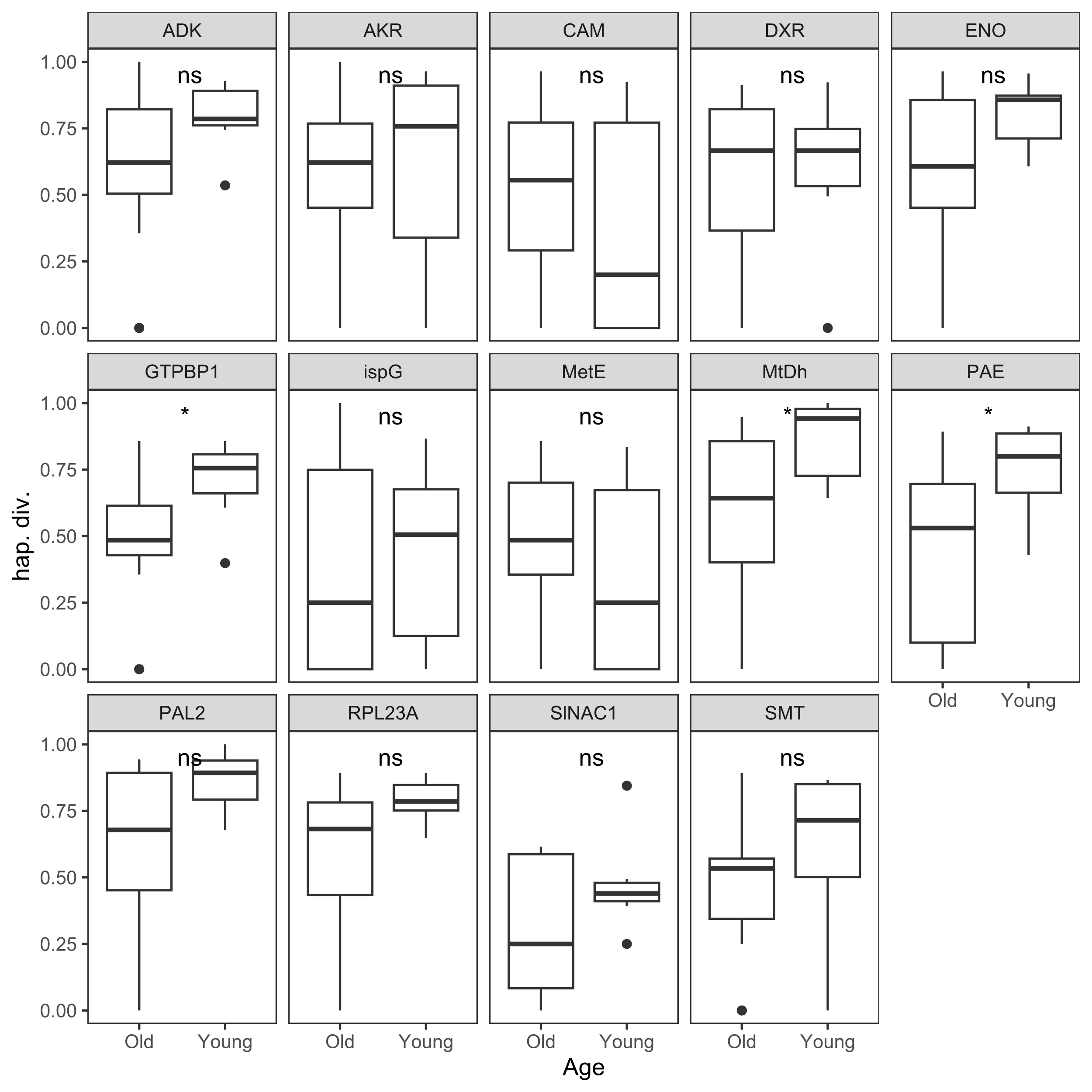

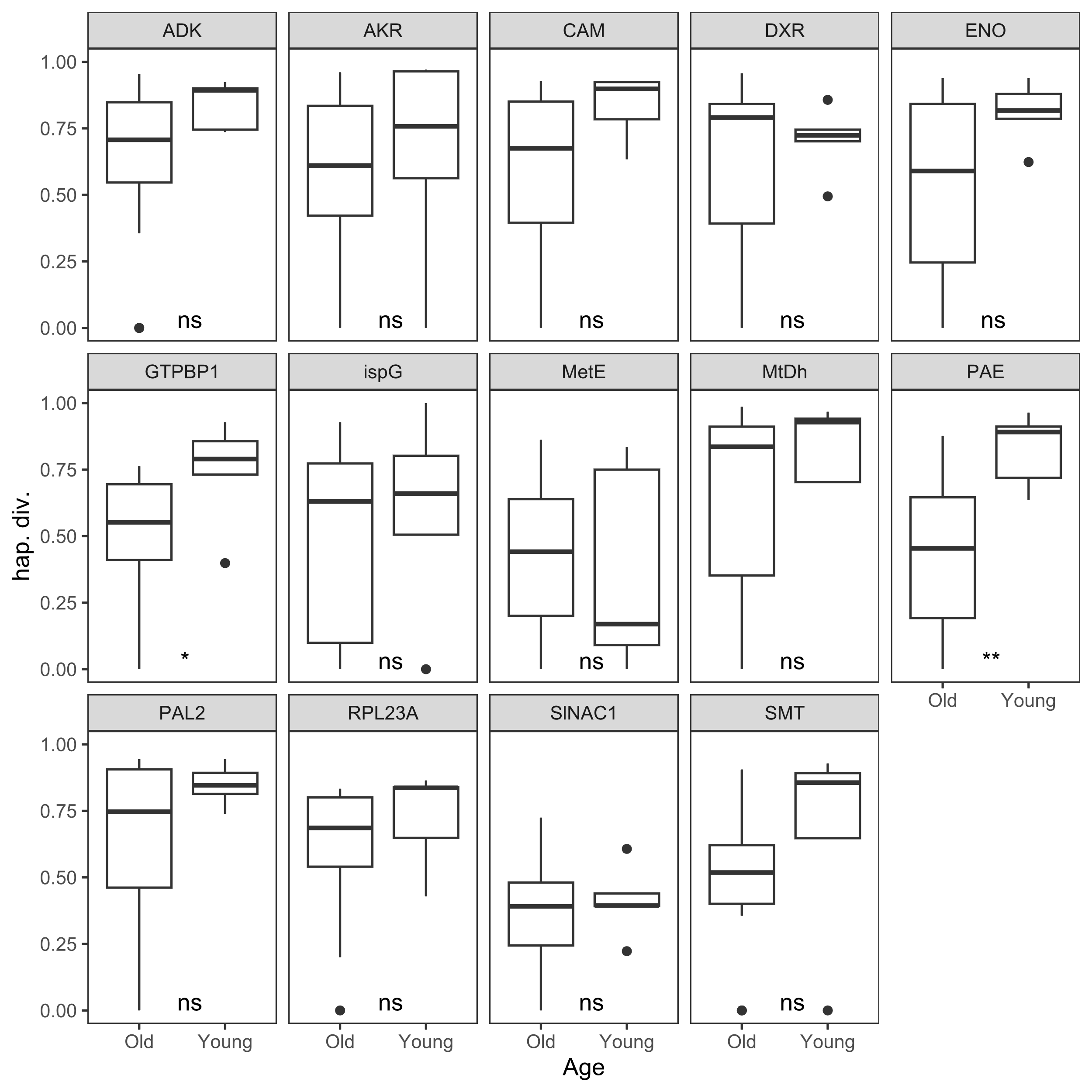

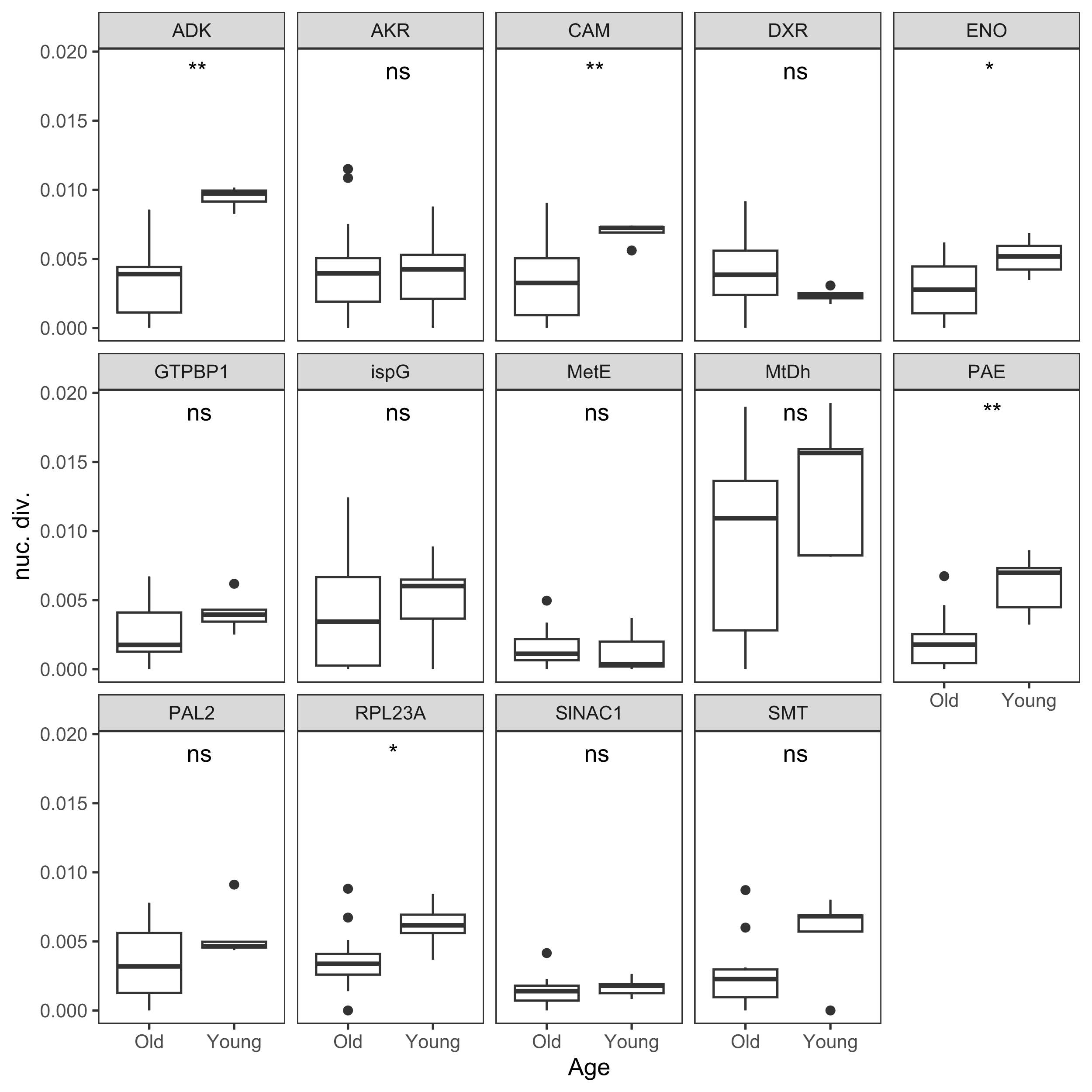


Supplementary Figure S4: Comparison of genetic diversity per species between older and younger islands estimated based on nucleotide (left) and haplotype (right) diversity. ns – nonsignificant difference (p ≥ 0.05), * significant difference (p < 0.05), ** significant difference (p < 0.01).


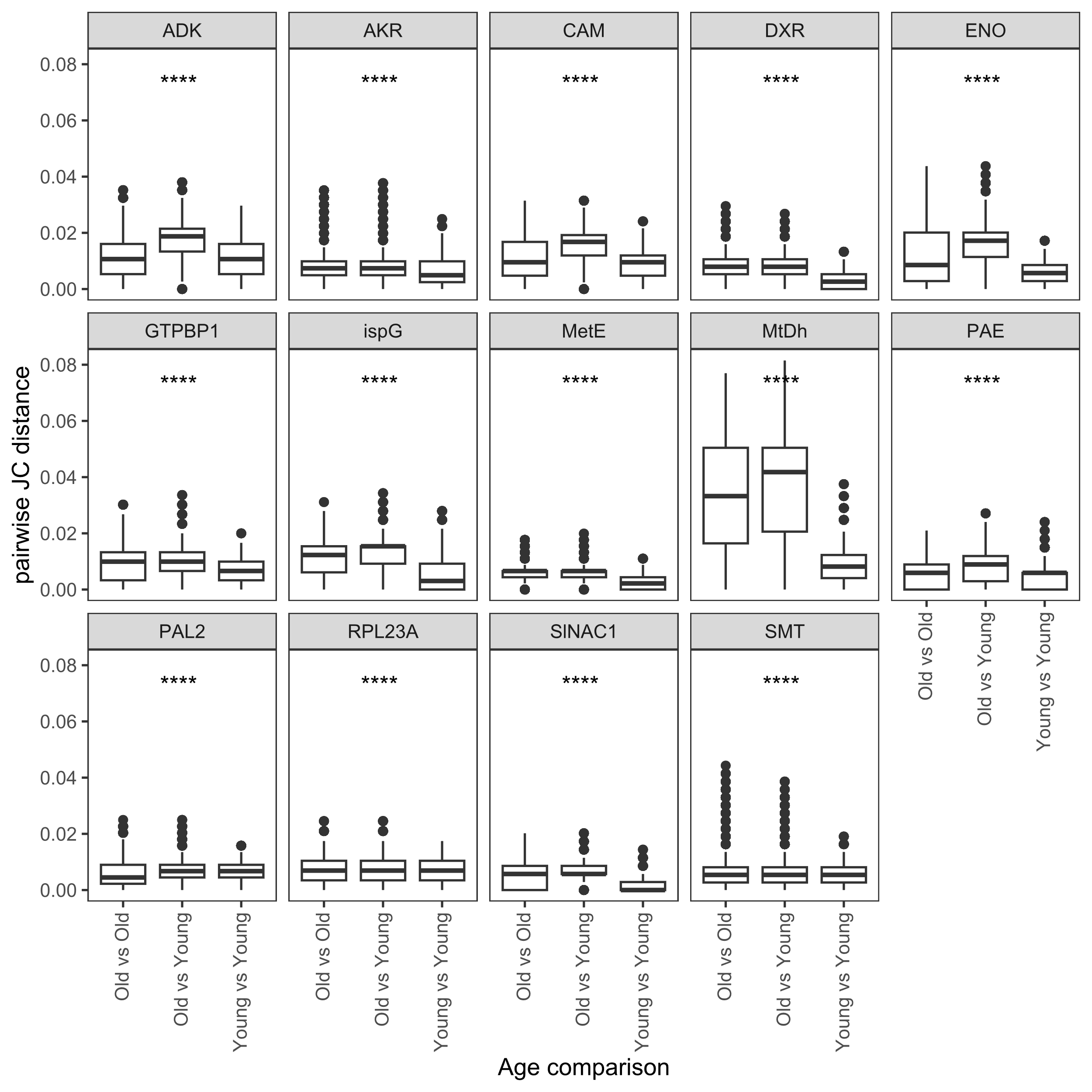


Supplementary Figure S5: Comparison of genetic differentiation per species between older and younger islands estimated based on pairwise sequence divergence. ns – nonsignificant difference (p ≥ 0.05), * significant difference (p < 0.05), ** significant difference (p < 0.01).


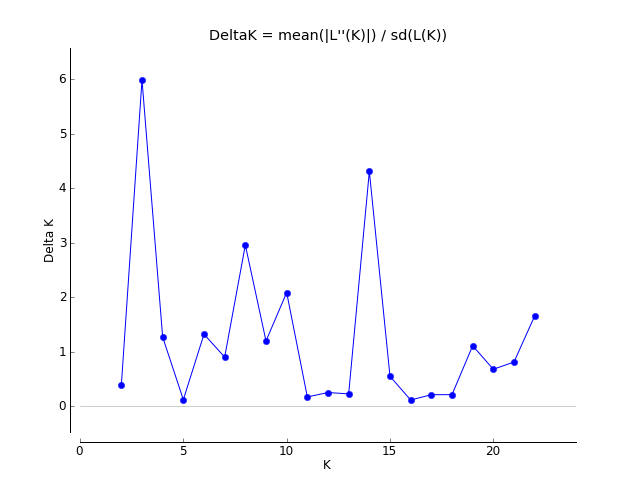

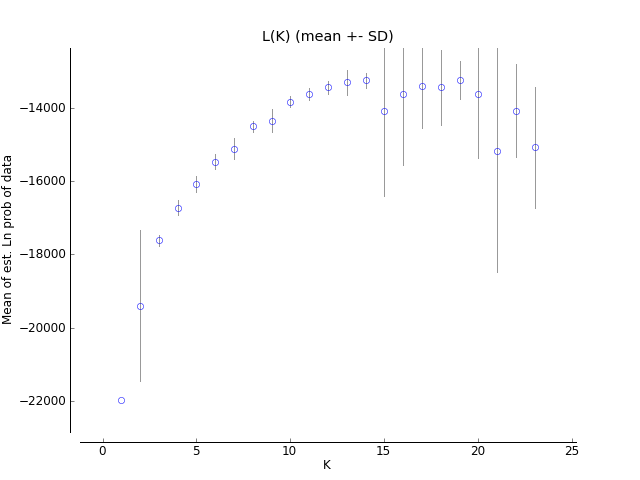

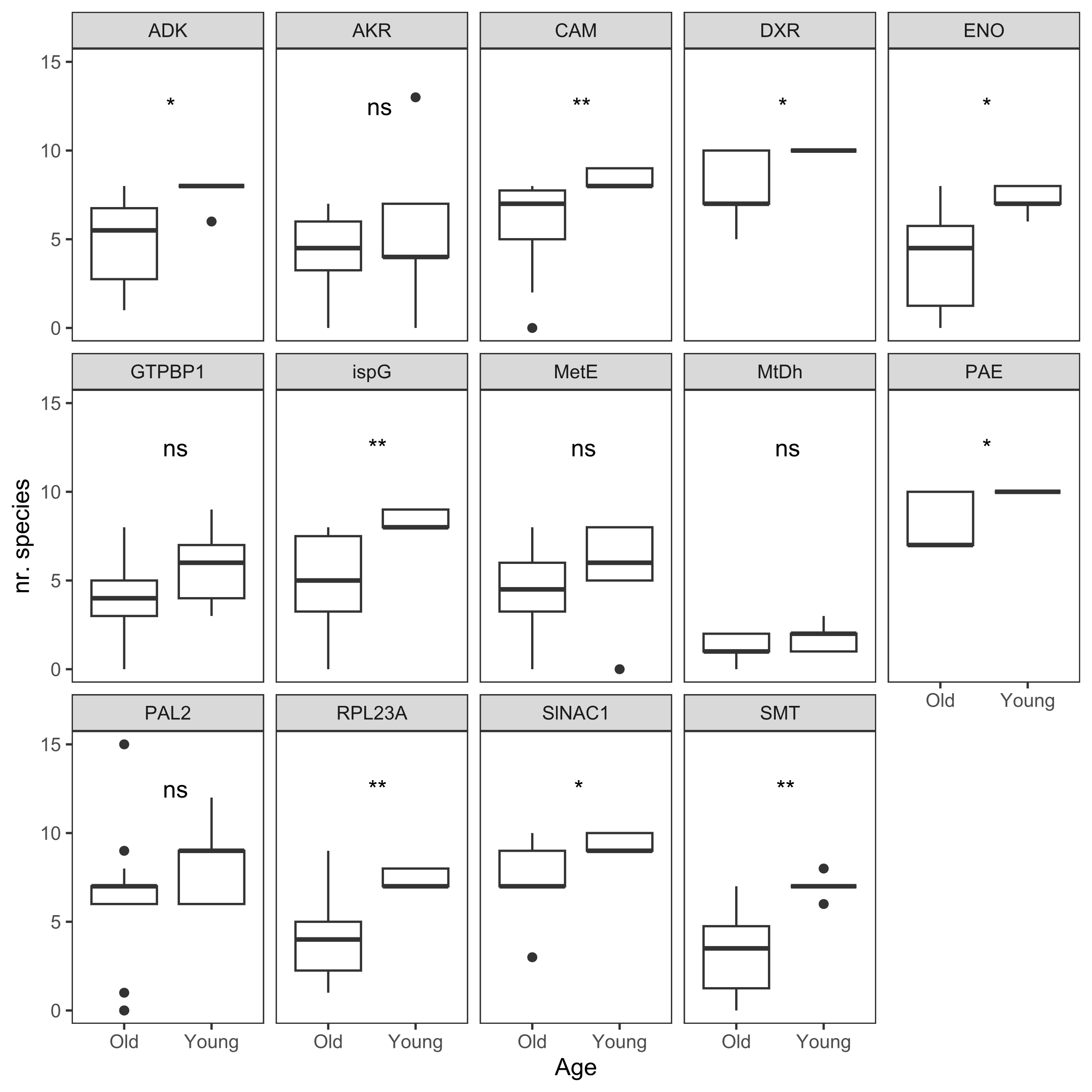
Supplementary Figure S6: Number of species sharing haplotypes for each of the included *Micromeria* taxa compared between older and younger islands for each marker. ns – nonsignificant difference (p ≥ 0.05), * significant difference (p < 0.05), ** significant difference (p < 0.01).

Supplementary Figure S7: DeltaK and mean likelihood per K value estimated with Structure Harvester.
